# Histone deacetylase HDAC7 restricts CD8 + T cell tumor infiltration and limits immunotherapy sensitivity in bladder cancer: reversal by pinocembrin

**DOI:** 10.1186/s13046-025-03585-3

**Published:** 2025-12-24

**Authors:** Jiancheng Lv, Kai Li, Jiatong Zhou, Ruixi Yu, Qiang Lu, Ben Liu

**Affiliations:** 1 Department of Urology, First Affiliated Hospital, School of Medicine, Zhejiang University, Hangzhou, Zhejiang China; 2 Department of Urology, The First Affiliated Hospital of Nanjing Medical University, Nanjing, Jiangsu China; 3 Department of Urology, The Second Affiliated Hospital of Nanjing Medical University, Nanjing, Jiangsu China; 4 Department of Urology, Jiangnan University Medical Center, Wuxi, 214000 China; 5 Cancer Center, Zhejiang University, Qingchun Road 79, Hangzhou, Zhejiang 310003 China

**Keywords:** BCa, HDAC7, CD8 + T cell, Tumor infiltration, Immunotherapy, Pinocembrin

## Abstract

**Background:**

The limited response rate and substantial interindividual variability in immunotherapy outcomes remain major barriers to improving prognosis in patients with bladder cancer (BCa). As central effectors of antitumor immunity, the extent of CD8 + T cell infiltration into tumors is a key determinant of immunotherapy response. Members of the histone deacetylase (HDAC) family play critical roles in modulating tumor immune evasion and sensitivity to immunotherapy, making HDAC inhibitors of clinical interest.

**Methods:**

A retrospective analysis was performed using data from the IMvigor210 clinical trial and follow-up data from patients with locally advanced BCa who received adjuvant immunotherapy at our center, assessing the association between HDAC1–11 expression and immunotherapy response. RNA sequencing, gene set enrichment analysis (GSEA), chromatin immunoprecipitation PCR (ChIP-PCR), co-immunoprecipitation (Co-IP), mass spectrometry, lysine site mutagenesis, RNA immunoprecipitation, and bioinformatics analysis were employed to outline the HDAC7–BTRC–SRSF7–CCL5 pathway. The immunoregulatory function of HDAC7 was evaluated using CD8 + T cell co-culture assays and tumor models in humanized NOG (HuNOG) mice. Virtual screening, MicroScale Thermophoresis (MST), and HDAC activity assays were conducted to identify potential HDAC7 specific inhibitor. The immunosensitizing effect of Pinocembrin on BCa immunotherapy was validated using a C57BL/6 mouse tumor-bearing model.

**Results:**

Among the HDAC family members, only HDAC7 expression was significantly associated with immunotherapy response. HDAC7 was overexpressed in BCa and correlated with poorer prognosis. Functional assays demonstrated that HDAC7 suppresses CD8 + T cell infiltration, thereby reducing sensitivity to PD-1 antibody treatment. Mechanistically, HDAC7 reduced acetylation at lysine 24 of the splicing regulator SRSF7, enhancing BTRC-mediated ubiquitination and degradation of SRSF7, which promoted the processing and expression of CCL5 mRNA-a chemokine essential for CD8 + T cell recruitment. Furthermore, Pinocembrin was identified as a selective HDAC7 inhibitor that restores CD8 + T cell infiltration and improves immunotherapy efficacy in BCa.

**Conclusions:**

HDAC7 represents a promising diagnostic and therapeutic target in BCa immunotherapy. Pinocembrin, as a specific HDAC7 inhibitor, holds potential as a combination therapy agent to improve immunotherapy response in BCa.

**Supplementary Information:**

The online version contains supplementary material available at 10.1186/s13046-025-03585-3.

## Introduction

Bladder cancer (BCa) is a significant global health challenge and ranks among the most prevalent malignancies of the genitourinary tract [1]. According to data from the National Cancer Center of China, an estimated 92,900 new BCa cases and approximately 41,400 deaths occurred in China in 2022 [2]. Standard treatments for BCa—including surgery, chemotherapy, and radiotherapy—are frequently limited by high recurrence rates and the frequent emergence of therapy resistance [3]. In recent years, immunotherapy has emerged as a promising approach for BCa management. Immune checkpoint inhibitors (ICIs), such as anti-PD-1/PD-L1 antibodies, have demonstrated efficacy in a subset of patients. However, overall response rates remain relatively low [4], and there are notable disparities in treatment efficacy among individuals [5]. Therefore, investigating the mechanisms underlying immunotherapy resistance and optimizing immunotherapy efficacy is of substantial clinical relevance.

CD8 + T cells are central to anti-tumor immunity, recognizing and eliminating tumor cells through their T-cell receptors (TCRs). Greater infiltration of CD8 + T cells within tumors correlates with more favorable prognosis in several cancers, including BCa [6]. Studies have demonstrated that higher densities of tumor-infiltrating CD8 + T cells are associated with improved overall survival and cancer-specific survival in BCa patients [7]. Increasing the infiltration of CD8 + T cells into tumors is crucial for enhancing immunotherapy response rates. Therefore, strategies that enhance the function and infiltration of CD8 + T cells in the tumor microenvironment (TME) represent important approaches to optimize therapeutic results in BCa immunotherapy.

Histone deacetylases (HDACs) are a family of enzymes that regulate gene expression by removing acetyl groups from histone proteins, resulting in chromatin condensation and gene silencing [8]. In addition, HDACs exert post-translational regulatory functions by catalyzing the deacetylation of non-histone proteins, thereby modulating protein stability [9]. HDACs are implicated in a range of cellular processes, including proliferation, differentiation, apoptosis, and immune regulation [10]. Aberrant expression and dysregulated activity of HDACs have been associated with tumorigenesis, progression, and immunotherapy sensitivity in various cancers [11, 12]. For example, HDAC3 has been shown to promote expression of the lung cancer lineage transcription factor NKX2-1, which drives progression and therapeutic resistance in non-small-cell lung cancer (NSCLC) [13]. HDAC3 inhibition can induce CXCL12 secretion and increase NK cell infiltration, providing an immunostimulatory strategy for T-cell lymphoma [14]. In contrast, HDAC5 negatively regulates PD-L1 expression, reducing the sensitivity of pancreatic cancer to immunotherapy [15]. HDAC7, a member of the HDAC family, has gained attention due to its potential roles in tumorigenesis and cancer progression [16]. For instance, HDAC7 is highly expressed in NSCLC and promotes proliferation and metastasis via activation of the β-catenin–FGF18 pathway [17]. HDAC7 also facilitates renal cancer progression by reprogramming branched-chain amino acid metabolism [18] and drives the malignant phenotype of lung adenocarcinoma (LUAD) by activating ZEB1 and c-MYC [19]. However, the roles and mechanisms of HDAC7 in BCa remain poorly characterized, and its function in tumor immune evasion and immunotherapy sensitivity is largely unexplored.

HDAC inhibitors (HDACis) have been developed as anticancer agents and have shown promising preclinical results [20]. Several HDACis, including vorinostat, romidepsin, and belinostat, have been approved for hematological malignancies [21]. However, their use in solid tumors, including BCa, is limited by toxicity and insufficient specificity [22]. Recent research has shifted toward the development of novel and highly specific HDAC inhibitors. For instance, the HDAC inhibitor SAHA enhances CD8 + T-cell-mediated antitumor activity via the HDAC1/JAK1/FGL1 axis [23]. Furthermore, Fusobacterium nucleatum increases immunotherapy sensitivity in colorectal cancer by producing butyric acid, which inhibits HDAC3/8 activity in CD8 + T cells [24]. Such studies highlight the potential for combining HDAC inhibitors with immunotherapy in cancer therapy.

The serine/arginine-rich splicing factor (SRSF) family is essential for alternative splicing, which generates protein diversity from a limited number of genes [25]. SRSF proteins recognize specific RNA sequences to promote or inhibit exon inclusion [25]. Dysregulation of SRSF family members is implicated in various cancers, including BCa [26]. For example, SRSF1 is highly expressed in BCa and promotes both tumor progression and cisplatin resistance via the HIF1A/BNIP3/mitophagy axis [27]. SRSF3 and SRSF9 have also been associated with prognosis and immune evasion in several malignancies [28, 29]. The SRSF family participates in modulating tumor immunotherapy responses; for example, SRSF10 induces immunotherapy resistance in hepatocellular carcinoma by promoting M2 macrophage polarization [30]. However, the role and mechanisms of SRSF7 in BCa remain to be fully elucidated.

Chemokine C-C motif ligand 5 (CCL5) is a chemokine with a critical role in recruiting and activating immune cells, including CD8 + T cells [31]. CCL5 signals through the chemokine receptor CCR5, which is expressed on CD8 + T cells [32]. By binding CCR5, CCL5—secreted by tumor or stromal cells—guides the migration of CD8 + T cells to sites of inflammation and tumor tissue. High tumor CCL5 expression is associated with greater CD8 + T cell infiltration and improved patient prognosis [33]. CCL5 has also emerged as a potential target to improve immunotherapy efficacy. For example, HSF1 impairs immune responses in breast cancer by inhibiting CCL5-mediated CD8 + T cell infiltration [34], and CCL5 is recognized as a promising biomarker to guide immunotherapy in ovarian cancer [35].

## Methods

### Retrospective analyses of clinical data

The correlation between the HDAC family (HDAC1–11) and the immunotherapy response rate in BCa patients was analyzed using the BEST website (https://rookieutopia.com/) and the IMvigor210 BCa immunotherapy cohort. IMvigor210 is an open-label, multicenter, single-arm phase II clinical trial that evaluated the efficacy and safety of Tecentriq for locally advanced or metastatic urothelial carcinoma (mUC) [36]. For each sample in the IMvigor210 cohort (responders vs. non-responders), we computed the Z-score of HDACs expression as (X − µ)/σ, where X is the raw expression value, µ the cohort-wide mean, and σ the standard deviation; this Z-score was taken as the normalized HDACs expression level. Differences between responders and non-responders were assessed with a two-tailed Student’s t-test, and *P* < 0.05 was considered statistically significant. In addition, we integrated clinical data from our center and selected patients with muscle-invasive bladder cancer (MIBC) who underwent radical cystectomy followed by adjuvant immunotherapy (in cases where chemotherapy was refused or not tolerated) as study participants. RNA and protein were extracted from patient tumor specimens; quantitative polymerase chain reaction (qPCR) and Western blot analysis were used to assess HDAC family expression in these samples. Postoperative patients were followed every three months to monitor survival and recurrence. Combined with long-term follow-up data, the correlation between HDAC family expression and overall prognosis was analyzed using the Kaplan–Meier method.

### Human tissue specimens and cell lines

Between 2019 and 2024, 40 paired BCa tissue samples were obtained from patients undergoing radical surgery at the First Affiliated Hospital of Nanjing Medical University. Patient survival ranged from 6 to 59.5 months, with follow-up periods extending from surgery to the detection of disease progression or recurrence. Approval for use of BCa tissues was granted by the hospital’s Ethics Committee, and written informed consent was obtained from all participants. Pathological review confirmed all tumor tissues as BCa; adjacent normal tissues from the same patients were verified as tumor-free. T24 (CVCL_0554), RT4 (CVCL_0036), 5637 (CVCL_0126), UMUC3 (CVCL_1783), J82 (CVCL_0359) and a normal bladder epithelial immortalised cell line SV-HUC (CVCL_3798) were obtained from the Type Culture Collection of the Chinese Academy of Sciences (Shanghai, China) in June, 2022. 253 J (CVCL_7935), BIU87 (CVCL_6881) and mouse bladder carcinoma cell line MB49 (CVCL_7076) were obtained from YaJi biological (Shanghai, China) in August, 2022. Cell identity was confirmed by short-tandem-repeat (STR) profiling, and absence of mycoplasma contamination was verified every six month.

### Cell culture and transfection

T24, UMUC3, and MB49 cell lines were cultured in Dulbecco’s modified Eagle’s medium (DMEM, Gibco, USA) containing 10% fetal bovine serum (FBS, BI, Israel) at 37 °C in a humidified atmosphere with 5% CO₂. Lentiviral vectors for HDAC7 knockdown and overexpression were obtained from HANBIO (Shanghai, China). When T24 or UMUC3 cells reached 50% confluence, they were transfected with HDAC7 knockdown (shHDAC7-1 and shHDAC7-2), negative control (shNC), HDAC7 overexpression, or vector control lentivirus. Transfected cells underwent dual puromycin selection at 5 and 10 µg/mL. Overexpression plasmids, mutation plasmids, and small interfering RNAs (siRNAs) targeting SRSF7, BTRC, and CCL5 were provided by GenePharma Co. (Shanghai, China). Transfection of T24 and UMUC3 cells was performed using the Lipofectamine 3000 kit (Invitrogen, USA) according to the manufacturer’s protocol. The siRNAs used in this study were listed in Table S1.

### RNA extraction and quantitative real time-PCR (qRT-PCR)

Total RNA was extracted from BCa tissues or cultured cells using TRIzol reagent (Invitrogen, USA). cDNA was synthesized from RNA using HiScript II reagent (Vazyme, China). Quantitative real-time PCR was performed with a SYBR pre-mix kit (Vazyme, Nanjing, China) on either the StepOne Plus real-time PCR system (Applied Biosystems, USA) or the LightCycler 480 (Roche, USA). Primers, listed in Supplemental Table S2, were synthesized by TsingKe (Nanjing, China).

### Protein isolation and western blot

Proteins were extracted from tissues and cells using RIPA buffer (Sigma, USA), and concentrations were determined using a bicinchoninic acid (BCA) kit (Beyotime, China). Proteins were separated by SDS-PAGE and transferred to polyvinylidene fluoride membranes. After blocking with 5% skim milk, membranes were incubated with primary antibodies (Abcam, USA) and secondary antibodies (Protech, USA). Following multiple washes, detection was achieved by chemiluminescence (Bio-Rad, USA) and analyzed using Image Lab software (Bio-Rad, USA).

### Enzyme-linked immunosorbent assay (ELISA)

The concentrations of human CCL5, granzyme B, and IFN-γ were measured using ELISA kits (FACs, Nanjing, China), following the manufacturer’s instructions.

### RNA sequencing

Transcriptome sequencing was performed on HDAC7 knockdown and control T24 cells by Allwegene Tech (Beijing, China) after total RNA extraction with TRIzol reagent (Invitrogen, USA).

### Chromatin Immunoprecipitation (ChIP) and ChIP-qPCR

BCa cells were crosslinked with 1% formaldehyde at 37 °C for 10 min. After lysis, chromatin was sheared by ultrasonication into fragments of 100–300 bp. The chromatin supernatant was incubated overnight at 4 °C with rotation and antibodies specific to H3K27ac, H3K9ac, or IgG. DNA was then eluted, purified, and quantified by RT-qPCR.

### Co-Immunoprecipitation (CO-IP) and mass spectrometry

BCa cells were lysed in buffer containing 20 mM Tris (pH 7.5), 150 mM NaCl, 1% Triton X-100, sodium pyrophosphate, β-glycerophosphate, EDTA, Na₃VO₄, leupeptin, and 1 mM PMSF on ice for 30 min. The lysate was centrifuged at 13,200 rpm at 4 °C for 15 min. The supernatant was incubated overnight at 4 °C with protein A/G agarose beads (Thermo Fisher Scientific, USA) and the relevant primary antibody. After six washes with lysis buffer, samples were subjected to Western blot or mass spectrometry.

### RNA immunoprecipitation (RIP)

Cells were lysed in IP lysis buffer, and RNA immunoprecipitation was conducted using the Magna RIP RNA-binding protein immunoprecipitation kit (Millipore, USA) according to the manufacturer’s protocol. Antibodies against SRSF7 (Abcam, USA) and control IgG (Millipore, USA) were used for immunoprecipitation. RNA from input and immunoprecipitated samples was isolated with TRIzol reagent (Invitrogen, USA), and the quantity of target RNA was determined by qRT-PCR and normalized to the input.

### CD8 + T cell culture and functional assay

Human peripheral blood mononuclear cells (PBMCs) were isolated from the venous blood of healthy volunteers using a PBMC isolation kit (FACs, Nanjing, China). CD8 + T cells were subsequently purified from PBMCs with CD8 microbeads (Miltenyi, Germany). The isolated CD8 + T cells were cultured in RPMI-1640 medium (Gibco, USA) and stimulated for 72 h with CD3 antibodies (2 µg/mL, Invitrogen, USA), CD28 antibodies (1 µg/mL, Invitrogen, USA), and interleukin-2 (IL-2, 5 ng/mL, R&D Systems, USA).

For chemotaxis assays, a 3 μm-pore Transwell insert (Corning, USA) was placed in a 24-well plate. Approximately 4 × 10⁵ activated CD8 + T cells were seeded into the upper chamber, and 1 × 10⁶ BCa cells were placed in the lower chamber. After 24 h of incubation, the number of CD8 + T cells that migrated to the lower chamber was determined by Cell Counting Kit-8 (CCK-8) assay. Following a 72-hour co-culture, supernatants were collected, and the concentrations of IFN-γ and granzyme B were measured with ELISA kits (FACs, Nanjing, China). After removing CD8 + T cells and cell debris, BCa cells were washed with phosphate-buffered saline (PBS). The viability of BCa cells was assessed at 570 nm using a microplate reader, and the remaining viable BCa cells were fixed with 4% paraformaldehyde and stained with 0.1% crystal violet.

### Cell proliferation and cloning formation assays

To assess BCa cell proliferation, 2,000 T24 or UMUC3 cells were seeded per well in 96-well plates. Cell viability was measured at 24, 48, 72, and 96 h using CCK-8 (Dojindo, Japan) and quantified with a microplate reader (Tecan, Switzerland) at 450 nm. For colony formation assays, 1,000 T24 or UMUC3 cells were seeded in each well of six-well plates. After 14 days of incubation, the cells were fixed with 4% paraformaldehyde and stained with 0.1% crystal violet. Colonies were visualized and quantified using ImageJ software (NIH, USA).

### Animal studies

NOG (NOD-scid IL2Rgnull) mice, which lack T and B cells and have severely impaired NK cell and macrophage function, were obtained from Shanghai Charles River Co. To generate humanized immune system mice (HuNOG mice; 5-week-old females, three per group), 1 × 10⁷ PBMCs from healthy donors were injected via the tail vein. Simultaneously, 1 × 10⁷ T24 cells (either HDAC7 or vector control) were implanted into the axilla. Peripheral blood was collected from the orbital sinus, and the proportion of human CD45-positive PBMCs was monitored weekly by flow cytometry with FITC-CD45 antibodies (Biolegend, USA). HuNOG mice were considered successfully humanized when CD45 positivity exceeded 50%. When tumor volumes reached approximately 100 mm³, experimental groups received PD1 antibodies (5 µg/g) intraperitoneally every other day for 2 weeks; control HuNOG mice received IgG antibodies. Tumor size was measured every 3 days from the first PD1 antibody injection. One month later, all HuNOG mice were euthanized, and tumor tissues were collected to determine volume and mass. Further immunohistochemical and flow cytometric analyses were performed to assess CD8 + T cell infiltration and anti-tumor activity in the tumor samples.

In C57BL/6 mice (5-week-old females, five per group), 1 × 10⁷ MB49 cells were implanted into the axilla. When tumor volume reached 100 mm³, mice received intraperitoneal injections of Pinocembrin (50 µg/g). Control mice were injected with saline. After 24 h, anti-PD1 antibodies (5 µg/g) were administered intraperitoneally, and treatment was maintained for 2 weeks. Following treatment, mice were monitored for an additional 2 weeks, then euthanized. Tumors were excised for volume and mass measurement, and subsequent immunohistochemical and flow cytometric analyses were performed to assess CD8 + T cell infiltration and function. All animal procedures were approved by the Animal Ethics Board of the First Affiliated Hospital, School of Medicine, Zhejiang University.

### Immunohistochemistry (IHC)

Tumors from HuNOG or C57BL/6 mice were formalin-fixed and paraffin-embedded. Section (4 μm thick) were prepared and mounted on slides. After deparaffinization and rehydration, antigen retrieval was performed by microwave heating. Endogenous peroxidase was quenched by immersion in 3% H₂O₂ for 10 min. Sections were incubated overnight at 4 °C with primary antibodies against CD8 and CD3 (Proteintech, USA). The following day, slides were incubated for 30 min at room temperature with horseradish peroxidase (HRP)-conjugated secondary antibodies. Stained sections were examined and imaged by light microscopy.

### Flow cytometric examination of the murine xenograft specimens

Tumor tissues were minced and digested with 2 mg/mL collagenase (Sigma, USA) in DMEM at 37 °C for 1 h. The resulting cell suspension was filtered through a 70 μm nylon mesh to remove debris. Cells were resuspended in PBS containing 2% bovine serum albumin (BSA) and stained with the indicated antibodies. After 15 min of incubation, cells were washed with PBS and analyzed by flow cytometry.

### Virtual screening of small-molecule compounds targeting HDAC7

Virtual screening was conducted using Schrödinger Maestro 12.8, with three-dimensional (3D) visualization performed in PyMol. The 3D structure of human HDAC7 (PDB ID: 3C10) was retrieved from the Protein Data Bank. The Protein Preparation Wizard module was used to add hydrogen atoms, remove water molecules, and delete the B and C chains. Missing atoms were added, and the protein structure was optimized using the OPLS2005 force field, applying a root-mean-square deviation (RMSD) threshold of 0.30 Å. The Receptor Grid Generation module was then used to create a grid file centered on the TSN ligand binding pocket. The two-dimensional (2D) structures of two compound libraries—HY-L001V MCE Bioactive Compound Library (22,300 compounds) and HY-L032V MCE Fragment Library (22,400 compounds)—were processed with the LigPrep module in Schrödinger, which added hydrogen atoms and optimized the structures to generate 3D conformations. The Virtual Screening Workflow module was employed to perform molecular docking. High-throughput virtual screening (HTVS) of the compound libraries was initially conducted using the Glide module. The top 15% of compounds, ranked by docking score, were selected for a second round of screening with the standard precision (SP) mode. The top 15% from this round were then further evaluated using extra precision (XP) mode. Final rankings were based on docking scores, and the highest-scoring compounds were selected for subsequent investigation. Pinocembrin 7-O-[3’’-O-galloyl-4’’,6’’-hexahydroxydiphenoyl] β-D-glucoside (hereafter referred to as Pinocembrin) was identified as the lead candidate due to its highest docking score.

### MicroScale thermophoresis (MST) experiment

To determine the binding affinity of HDAC7 with Pinocembrin, microscale thermophoresis (MST) was performed using Monolith NT.115 (NanoTemper Technologies GmbH, Munich, Germany) and standard capillaries. HDAC7 recombinant protein was obtained from SIGNALCHEM (cat. H89-31G), and Pinocembrin was obtained from the MedChemExpress (CAS: 205370-59−8). The HDAC7 recombinant protein was labeled with the fluorescent dye Red-NHS using NanoTemper’s labeling kit MO-L011 Monolith™ according to the manufacturer’s instructions. A fluorophore-to-protein molar ratio of 3.3 was used during labeling. After buffer exchange into PBST (10 mM PBS, 0.05% Tween-20, pH 7.4), 50 nM labeled HDAC7 was incubated with a 16-point two-fold serial dilution of the ligand ranging from 3.13 µM to 0.38 nM in TNT buffer (100 mM Tris-HCl, 1.5 M NaCl, 0.5% Tween-20, pH 7.8–8.2) with 5% DMSO. Measurements were performed at 25 °C. The fluorescence signal was normalized, and the KD value was determined by fitting the binding curve using MO Affinity Analysis v2.3 software (NanoTemper).

### HDAC activity assay

HDAC7 was either knocked down or overexpressed in T24 and MB49 cells, which were then treated with increasing concentrations of Pinocembrin (10, 50, 100, 200 µM) for 48 h. After removing the supernatant and washing with PBS, cells were lysed, and lysates were collected. HDAC activity was measured using an HDAC Activity Assay Kit (Abcam, USA), demonstrating that Pinocembrin specifically inhibits HDAC7 activity.

### Statistical analyses

Statistical analyses were conducted using SPSS software (IBM, version 22.0). Data are presented as mean ± standard deviation (SD). Student’s t-test and one-way analysis of variance (ANOVA) were used to assess differences between groups. A two-sided P value < 0.05 was considered statistically significant. Survival analysis was performed using the Kaplan–Meier method, with the log-rank test applied to compare survival differences among groups. All experiments were conducted in triplicate to ensure data reliability.

## Results

### Retrospective clinical analyses reveals that HDAC7 correlates with immunotherapy response in BCa and is highly expressed in the disease

**Fig. 1:**
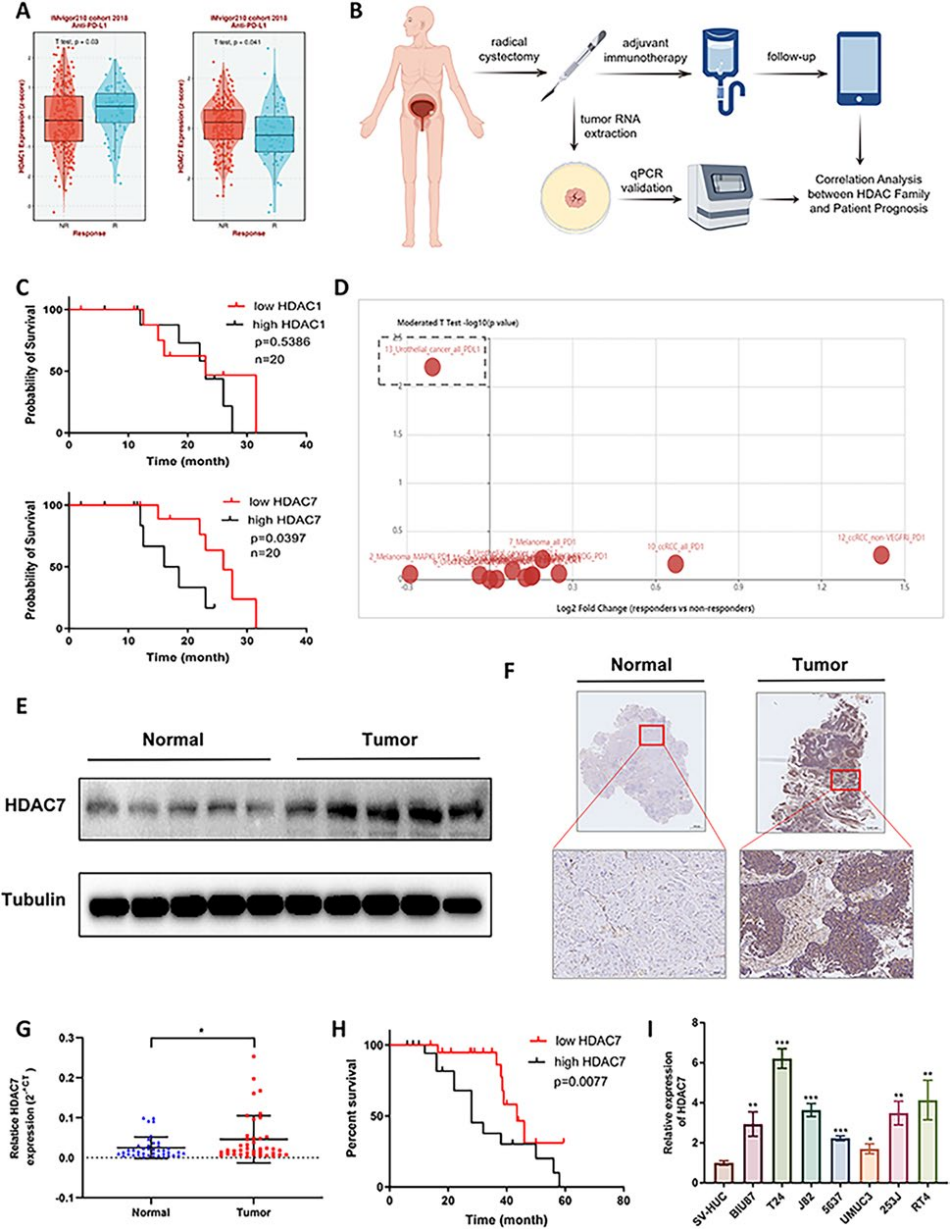
Retrospective clinical analysis indicates that HDAC7 is associated with the immunotherapy response in BCa.** A** Retrospective analysis of the IMvigor210 cohort identified HDAC1 and HDAC7 as BCa immunotherapy response-associated HDACs. **B** Flowchart illustrating the screening and follow-up of BCa patients who received adjuvant immunotherapy at our center, with analysis of the correlation between HDAC1 or HDAC7 expression and immunotherapy response. **C** Correlation analysis of HDAC1 or HDAC7 expression with the prognosis of BCa patients receiving adjuvant immunotherapy (*n* = 20). **D** Querying the TISIDB database revealed a negative correlation between HDAC7 expression and immunotherapy response in BCa. **E** Western blot analysis of HDAC7 expression in BCa tissues and adjacent non-tumor tissues. **F** IHC of HDAC7 expression in BCa tissues and adjacent non-tumor tissues. **G** qRT-PCR analysis of HDAC7 expression in 40 paired BCa tissues and adjacent non-tumor tissues (*P < 0.05). **H** Correlation between HDAC7 expression and overall prognosis in 40 BCa patients. **I** qRT-PCR analysis of HDAC7 expression in 7 BCa cell lines and one normal urothelial cell line (*P < 0.05, **P < 0.01, ***P < 0.001). Data are mean ± SD, *n* = 3

To identify HDAC subtypes associated with immunotherapy sensitivity in BCa, we analyzed the correlation between HDAC family genes (HDAC1–11) and immunotherapy response rates using the IMvigor210 BCa immunotherapy cohort. HDAC1 expression showed a positive correlation with BCa immunotherapy response, whereas HDAC7 was negatively correlated (Fig. 1 A, Figure S1). Incorporating follow-up data from our center, we selected MIBC patients who underwent radical cystectomy and received adjuvant immunotherapy (having refused or not tolerated chemotherapy) for further study. We examined HDAC1 and HDAC7 expression in postoperative tumor specimens and assessed their association with overall prognosis (Fig. 1B). High HDAC7 expression was significantly associated with poor prognosis, whereas HDAC1 did not demonstrate such a correlation (Fig. 1 C). Analysis via the TISIDB database (http://cis.hku.hk/TISIDB/) further confirmed that HDAC7 expression is negatively correlated with immunotherapy response in BCa patients (Fig. 1D, Table S3). We validated HDAC7 expression in 40 paired BCa tissues, finding that HDAC7 was significantly upregulated in tumor tissues compared with adjacent normal tissues at both mRNA and protein levels (Fig. 1E–G). Kaplan–Meier analysis showed that higher HDAC7 expression was associated with worse overall survival (Fig. 1H). Additionally, HDAC7 was found to be upregulated in seven BCa cell lines compared with SV-HUC cells (Fig. 1I). Analysis of The Cancer Genome Atlas (TCGA) data revealed that HDAC7 expression was elevated in ten tumor types, including BCa, compared to normal tissues (Figure S2A). Overall, HDAC7 was significantly upregulated in tumor tissues relative to adjacent normal tissues (Figure S2B), with the highest expression observed in thymoma (THYM) and diffuse large B-cell lymphoma (DLBC). Among 33 tumor types, HDAC7 expression in BCa ranked 13th (Figure S2C).

### *In vitro* assays reveal HDAC7 inhibits the chemotaxis and anti-tumor activity of CD8 + T cells in BCa

**Fig. 2:**
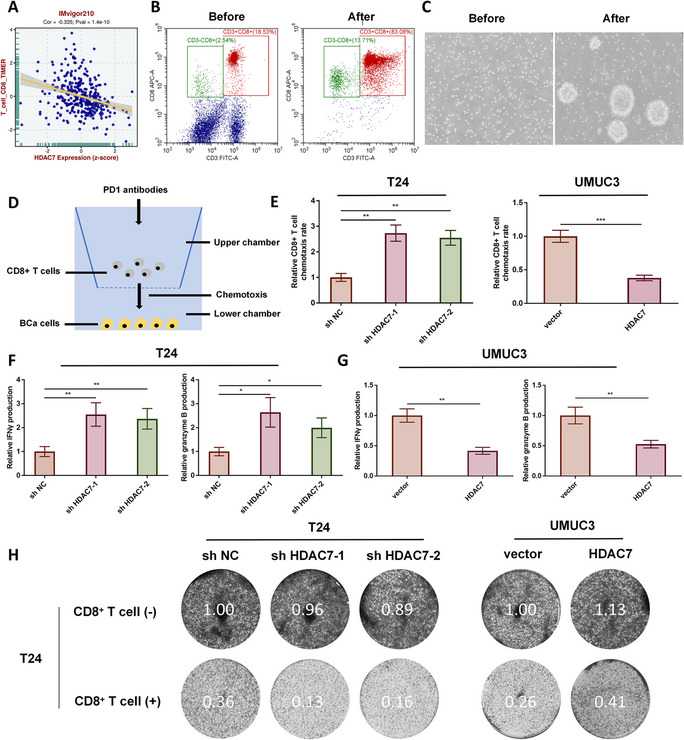
HDAC7 inhibits CD8 + T cell chemotaxis and anti-tumor activity in vitro. **A** Analysis of IMvigor210 cohort data shows negative correlation between HDAC7 expression and CD8 + T cell chemotaxis. **B** Flow cytometry confirms the efficiency of CD8 + T cell sorting using CD3 and CD8 antibodies. **C** Microscopy of CD8 + T cell morphology before and after activation. **D** Schematic of the co-culture model. **E** In vitro co-culture shows that HDAC7 inhibits CD8 + T cell chemotaxis (**P < 0.01, ***P < 0.001). **F** HDAC7 knockdown in T24 cells increases IFN-γ and granzyme B production by CD8 + T cells (*P < 0.05, **P < 0.01). (G) HDAC7 overexpression in UMUC3 cells inhibits IFN-γ and granzyme B production by CD8 + T cells (**P < 0.01). **H** CD8 + T cell cytotoxicity is enhanced when co-cultured with HDAC7 knockdown T24 cells and reduced when co-cultured with HDAC7-overexpressing UMUC3 cells. Data are mean ± SD, *n* = 3

IMvigor210 clinical data and TIMER analysis indicated that HDAC7 expression is negatively correlated with CD8 + T cell infiltration in BCa (Fig. 2 A). CD8 + T cells were isolated from PBMCs using CD8 microbeads, and isolation efficiency was confirmed by flow cytometry, with more than 80% of cells being CD3 and CD8 double-positive (Fig. 2B). After 72 h of activation, CD8 + T cells displayed marked expansion and clustering (Fig. 2 C). A two-chamber co-culture model was established to assess the effect of HDAC7 on CD8 + T cell chemotaxis and function (Fig. 2D). Knockdown of HDAC7 in T24 cells increased migration of activated CD8 + T cells, whereas HDAC7 overexpression in UMUC3 cells reduced CD8 + T cell migration (Fig. 2E).

Following 72-hour co-culture, ELISA revealed that HDAC7 knockdown in T24 cells increased IFN-γ and granzyme B secretion by CD8 + T cells, whereas HDAC7 overexpression in UMUC3 cells suppressed their production (Fig. 2F–G). The anti-tumor activity of CD8 + T cells was significantly enhanced by co-culture with HDAC7 knockdown T24 cells and reduced by co-culture with HDAC7-overexpressing UMUC3 cells (Fig. 2H). These results indicate that HDAC7 inhibits both the chemotactic and anti-tumor functions of CD8 + T cells in the BCa TME. Furthermore, CCK8 and colony formation assays demonstrated that HDAC7 promotes proliferation of T24 and UMUC3 BCa cells (Figure S3).

### *In vivo* assays demonstrate HDAC7 inhibits CD8 + T cell tumor infiltration and activity, reducing immunotherapy efficacy in BCa

**Fig. 3:**
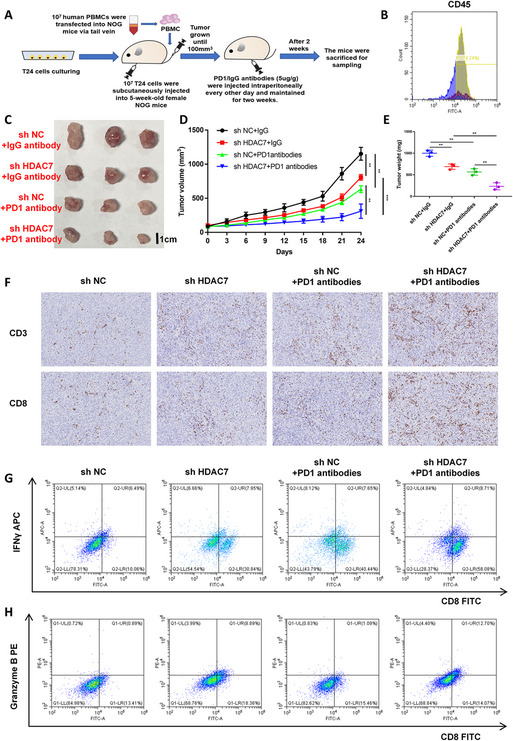
HDAC7 inhibits CD8 + T cell chemotaxis and immunotherapy sensitivity in BCa in vivo. **A** Schematic of the HuNOG mouse model and experimental design for studying HDAC7’s effect on CD8 + T cell chemotaxis and immunotherapy response. **B** Flow cytometry analysis of human CD45 positivity in HuNOG mice. **C** Representative tumor images from HuNOG mice injected with HDAC7 knockdown or control T24 cells and treated with PD-1 or IgG antibodies (*n* = 3). **D** Tumor volume in HuNOG mice measured every 3 days (**P < 0.01, ***P < 0.001). **E** Tumor weight assessment in HuNOG mice (**P < 0.01). **F** IHC analysis of CD3 and CD8 in tumors from HuNOG mice, showing increased CD8 + T cell infiltration with HDAC7 knockdown. **G**–**H.** Flow cytometry of CD8, IFN-γ, and granzyme B in tumor tissues, confirming that HDAC7 knockdown enhances CD8 + T cell infiltration and anti-tumor activity. Data are mean ± SD, *n* = 3

To investigate the effect of HDAC7 on CD8 + T cell anti-tumor function and immunotherapy sensitivity in vivo, we employed the HuNOG mouse model (Fig. 3A). Successful humanization of the mice was confirmed by flow cytometry, which detected over 70% human CD45-positive cells in peripheral blood (Fig. 3B). HuNOG mice were inoculated with either HDAC7 knockdown or control T24 cells and assigned to four groups: shHDAC7 + PD-1 antibody, shNC + PD-1 antibody, shHDAC7 + IgG, and shNC + IgG. Four weeks after PD-1 antibody administration, mice were euthanized and tumor samples collected (Fig. 3 C). Tumor volume and weight measurements indicated that HDAC7 knockdown significantly suppressed BCa growth and increased immunotherapy sensitivity in HuNOG mice (Fig. 3D–E). Immunohistochemical staining demonstrated increased CD8 and CD3 positivity in tumors with HDAC7 knockdown, indicating enhanced CD8 + T cell infiltration (Fig. 3 F). Flow cytometric analysis confirmed that HDAC7 knockdown both increased CD8 + T cell infiltration and improved their capacity to secrete IFN-γ and granzyme B in BCa tumors (Fig. 3G).

### HDAC7 is negatively associated with CD8 + T cell tumor infiltration through attenuation of CCL5 expression

**Fig. 4:**
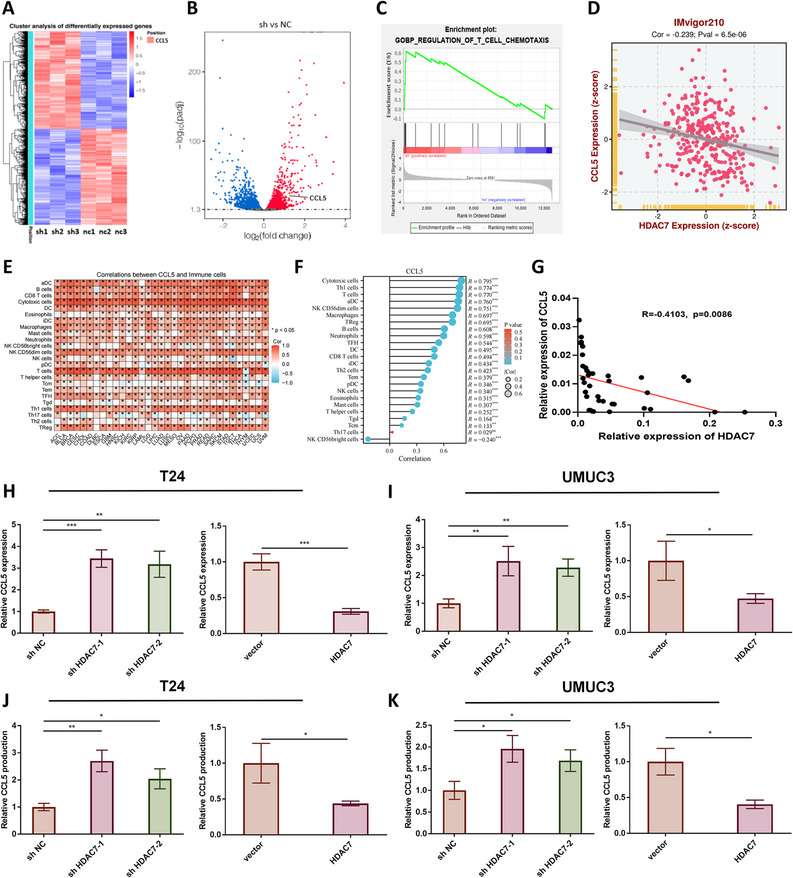
HDAC7 inhibits CCL5 expression and impedes CD8 + T cell infiltration in BCa. **A** Heat map of mRNA sequencing in three pairs of HDAC7 knockdown and control T24 cells. **B** Volcano plot of mRNA sequencing showing differentially expressed genes, including CCL5. **C** GSEA indicates enrichment of differentially expressed genes in the T cell chemotaxis pathway. **D** Analysis of the IMvigor210 cohort shows a negative correlation between HDAC7 and CCL5 expression. **E**–**F** Correlation between CCL5 expression and immune cell infiltration across cancers. **G** Pearson correlation between HDAC7 and CCL5 expression in 40 BCa tissues. **H**–**I** qRT-PCR analysis in T24 and UMUC3 cells demonstrates that HDAC7 knockdown upregulates, whereas overexpression downregulates, CCL5 expression (*P < 0.05, **P < 0.01, ***P < 0.001). **J**–**K** ELISA shows that HDAC7 inhibits CCL5 protein expression (*P < 0.05, **P < 0.01). Data are mean ± SD, *n* = 3

Transcriptome sequencing was performed on three sets of HDAC7 knockdown and control T24 cells. Differentially expressed genes were identified, and gene set enrichment analysis (GSEA) was conducted. A total of 2,539 genes were upregulated (including CCL5), and 2,498 genes were downregulated in HDAC7 knockdown cells compared to controls (Fig. 4A–B). GSEA indicated a negative association between HDAC7 expression and the T cell chemotaxis pathway (Fig. 4 C). Using IMvigor210 clinical data, we validated that HDAC7 expression is negatively correlated with CCL5 expression in BCa (Fig. 4D). CIBERSORT analysis of TCGA data demonstrated that CCL5 expression is positively correlated with infiltration of multiple immune cell types, including CD8 + T cells (Fig. 4E–F). In 40 BCa tissue samples, Pearson correlation analysis confirmed a negative association between CCL5 and HDAC7 expression (Fig. 4G). The effect of HDAC7 on CCL5 expression was verified by qRT-PCR and ELISA. HDAC7 knockdown increased CCL5 mRNA and protein levels in T24 and UMUC3 cells, whereas HDAC7 overexpression reduced CCL5 expression (Fig. 4H–K). To confirm that HDAC7 modulates CD8 + T cell chemotaxis via CCL5, we performed simultaneous knockdown of CCL5 in HDAC7 knockdown T24 and UMUC3 cells. In vitro co-culture experiments showed that the enhanced chemotactic ability of CD8 + T cells resulting from HDAC7 knockdown was reversed by CCL5 knockdown (Figure S4).

### HDAC7 negatively regulates SRSF7 through non-histone deacetylation, thereby inhibiting CCL5 expression

**Fig. 5:**
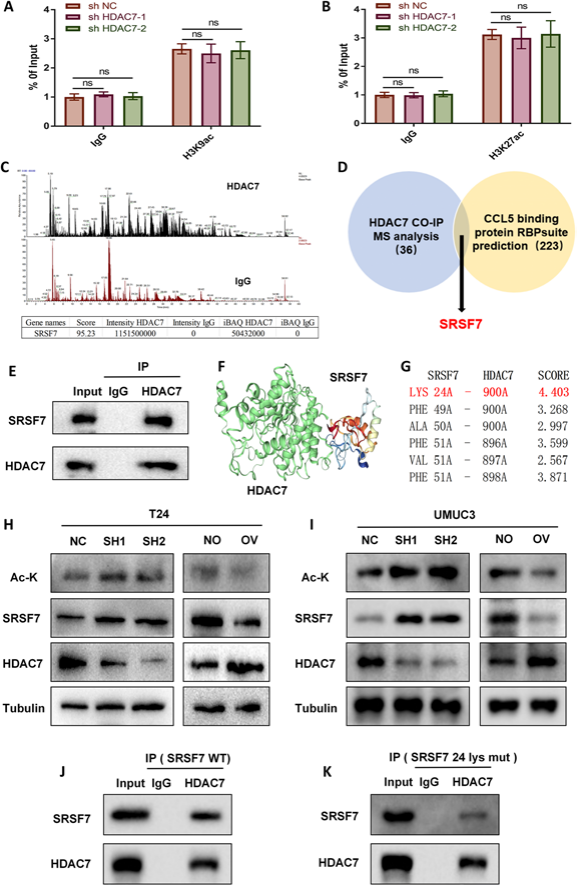
HDAC7 targets SRSF7 via non-histone deacetylation to regulate CCL5 expression. **A-B** ChIP-qPCR with H3K9ac and H3K27ac antibodies examines HDAC7’s effect on acetylation in the CCL5 promoter region. **C** CO-IP and mass spectrometry identify HDAC7 binding to SRSF7. **D** Integration of mass spectrometry and RBPsuite predictions identifies SRSF7 as the mediator of HDAC7-regulated CCL5 expression. **E** CO-IP confirms HDAC7–SRSF7 interaction. **F**–**G** HDOCK analysis reveals HDAC7 binding to SRSF7 at Lys24, a potential deacetylation site. **H**–**I** Western blot analysis of the effects of HDAC7 on SRSF7 acetylation (Lys24) and protein expression in T24 and UMUC3 cells. **J**–**K** CO-IP analysis of how SRSF7 Lys24 mutation affects HDAC7–SRSF7 binding in T24 cells. Data are mean ± SD, *n* = 3

HDAC7, a key histone deacetylase, removes acetyl groups from lysine residues on histones and suppresses gene transcription [37]. This mechanism predominantly acts at two promoter sites: H3K9ac and H3K27ac [38]. Using ChIP-qPCR, we investigated whether HDAC7 regulates histone acetylation at the CCL5 promoter. Unexpectedly, HDAC7 knockdown did not alter H3K9ac or H3K27ac levels in the CCL5 promoter region (Fig. 5A–B). We therefore hypothesized that HDAC7 regulates CCL5 expression through its non-HDAC activity. CO-IP assays and mass spectrometry revealed that HDAC7 does not directly bind CCL5 but interacts with several RNA-binding proteins (RBPs) (Fig. 5 C). Screening potential CCL5-binding RBPs using the RBPsuite database identified SRSF7 as a key mediator (Fig. 5D). CO-IP with HDAC7 antibodies confirmed that HDAC7 binds SRSF7 (Fig. 5E). HDOCK database analysis indicated that lysine 24 (Lys24) of SRSF7 is a likely deacetylation site targeted by HDAC7 (Fig. 5F–G). qPCR analysis showed that HDAC7 does not affect SRSF7 mRNA levels (Figure S5). However, Western blot assays demonstrated that HDAC7 knockdown in T24 and UMUC3 cells increases SRSF7 expression and acetylation at Lys24, whereas HDAC7 overexpression reduces SRSF7 expression and acetylation at this site (Fig. 5H–I). To confirm that HDAC7 binds and deacetylates SRSF7 at Lys24, we constructed SRSF7 Lys24 mutant (Lys→Ala) and wild-type plasmids, which were transfected into T24 cells. CO-IP and Western blot analyses showed that HDAC7 strongly binds wild-type SRSF7 but shows reduced binding to the Lys24 mutant (Fig. 5J–K).

### HDAC7 deacetylates SRSF7 Lys24, promoting btrc-mediated ubiquitination and degradation of SRSF7

**Fig. 6:**
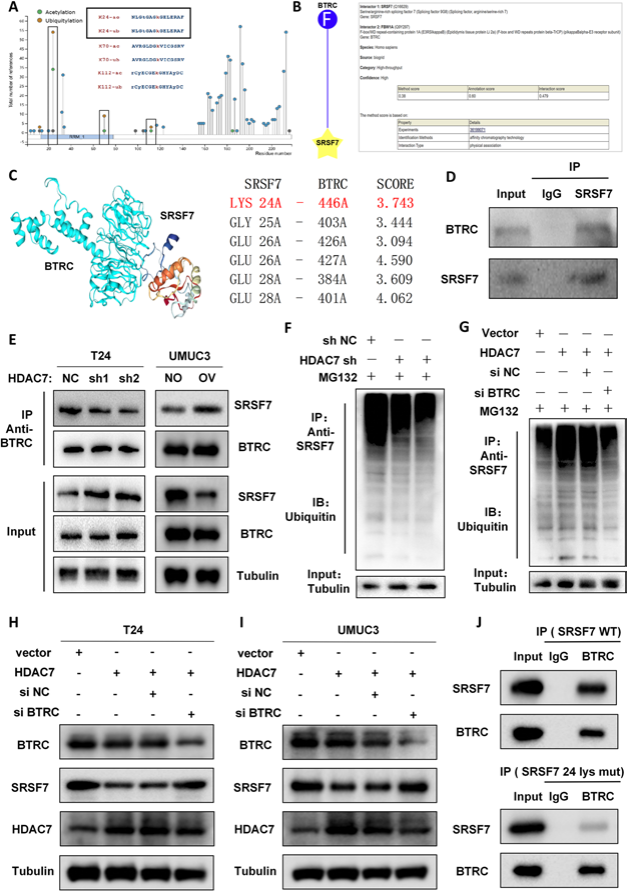
HDAC7 deacetylates SRSF7, promoting BTRC-induced ubiquitination and degradation of SRSF7.** A** PhosphoSitePlus analysis predicts acetylation and ubiquitination of SRSF7 at Lys24. **B**–**C** Ubibrowser and HDOCK predict BTRC binding to SRSF7 at Lys24. **D** CO-IP confirms BTRC–SRSF7 interaction. **E** CO-IP shows that HDAC7 knockdown promotes SRSF7 expression and reduces BTRC-SRSF7 binding in T24 cells. CO-IP shows that HDAC7 overexpression inhibits SRSF7 expression and increases BTRC-SRSF7 binding in UMUC3 cells. **F** CO-IP reveals that HDAC7 knockdown inhibits SRSF7 ubiquitination in T24 cells. **G** In T24 cells, HDAC7 overexpression increases SRSF7 ubiquitination, which is reduced by concurrent BTRC knockdown. **H**–**I** Western blot analysis shows that HDAC7 overexpression inhibits SRSF7 expression, which is restored by BTRC knockdown in T24 and UMUC3 cells. CO-IP analysis of SRSF7 Lys24 mutation on BTRC–SRSF7 binding in T24 cells. Data are mean ± SD, *n* = 3

Deacetylation by the HDAC family can promote target protein ubiquitination and proteasomal degradation [39, 40]. PhosphoSitePlus database analysis predicted that SRSF7 Lys24 undergoes both acetylation and ubiquitination (Fig. 6 A). Further database analyses (Ubibrowser and HDOCK) suggested that the E3 ubiquitin ligase BTRC binds SRSF7 at Lys24 (Fig. 6B–C), indicating that HDAC7-mediated deacetylation may enhance BTRC-dependent SRSF7 ubiquitination and degradation. CO-IP confirmed the BTRC–SRSF7 interaction (Fig. 6D). Additional CO-IP assays showed that HDAC7 knockdown reduces BTRC-bound SRSF7 and elevates SRSF7 expression in T24 cells, whereas HDAC7 overexpression increases BTRC–SRSF7 binding and decreases SRSF7 levels in UMUC3 cells (Fig. 6E). CO-IP with SRSF7 antibodies revealed that HDAC7 knockdown decreases ubiquitin bound to SRSF7 (Fig. 6 F, Figure S6), whereas HDAC7 overexpression increases SRSF7 ubiquitination; however, BTRC knockdown in HDAC7-overexpressing cells reduces SRSF7 ubiquitination (Fig. 6G). Western blot analysis confirmed that BTRC knockdown restores SRSF7 expression in HDAC7-overexpressing BCa cells (Fig. 6H–I). Furthermore, in cells transfected with SRSF7 Lys24 mutant plasmids, the amount of BTRC-bound SRSF7 was significantly reduced compared to wild-type (Fig. 6 J), indicating that both HDAC7 and BTRC interact with SRSF7 at Lys24.

### SRSF7 inhibits exon skipping of CCL5, promoting Mrna maturation

**Fig. 7:**
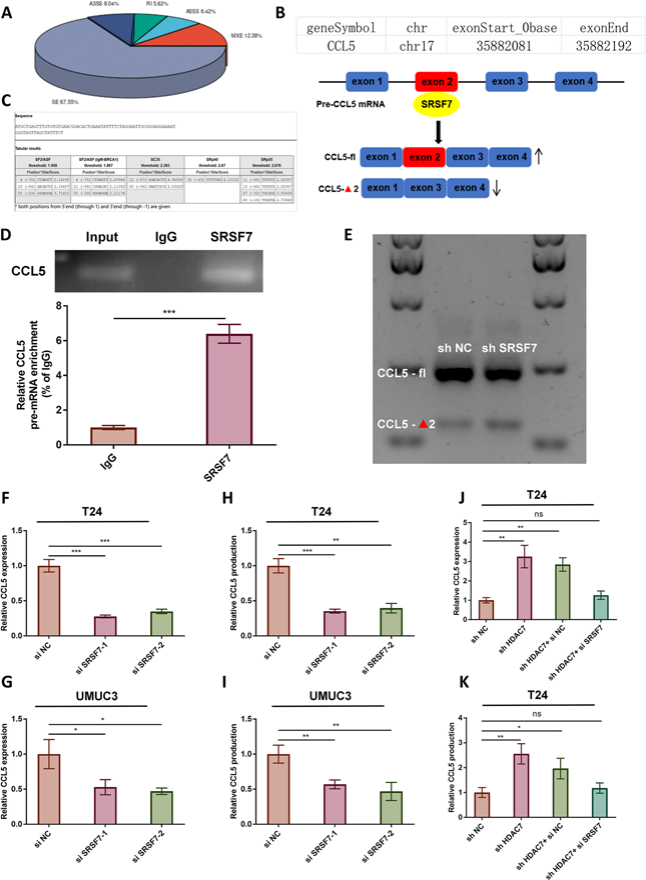
SRSF7 inhibits exon skipping of CCL5 pre-mRNA, thereby promoting mRNA maturation.**A** Analysis of alternative splicing (AS-Novel) in transcriptome sequencing data showed that exon skipping (ES) events accounted for 67.55% of all events. **B** AS-Novel analysis identified exon 2 (E2) skipping in CCL5. **C** ESE finder 3.0 predicted that CCL5 E2 contains exonic splicing enhancer (ESE) regions. **D** RIP assay confirmed the binding between SRSF7 and CCL5 pre-mRNA (***P < 0.001). **E** Primers spanning E2 and targeting E2-skipping were designed; PCR amplification and agarose gel electrophoresis were used to assess changes in CCL5 alternative splicing following SRSF7 knockdown in T24 cells. **F**–**G** qRT-PCR showed that SRSF7 knockdown inhibited CCL5 expression in T24 and UMUC3 cells (*P < 0.05, ***P < 0.001). **H**–**I** ELISA showed reduced CCL5 protein production following SRSF7 knockdown in T24 and UMUC3 cells (**P < 0.01, ***P < 0.001). **J**–**K** qRT-PCR and ELISA-based rescue experiments demonstrated that HDAC7 knockdown increases CCL5 expression, which is repressed when SRSF7 is simultaneously knocked down in T24 cells (*P < 0.05, **P < 0.01). Data are mean ± SD, *n* = 3

To clarify how SRSF7 regulates CCL5, we performed differential alternative splicing analysis using transcriptome sequencing data. Among alternative splicing events, exon skipping predominated, including skipping of CCL5 exon 2 (E2) (Fig. 7A–B). Exon skipping refers to splicing out an exon and its flanking introns, preventing its inclusion in mature mRNA [41]. SRSF proteins have been shown to reduce exon skipping and promote pre-mRNA splicing by binding to exon splicing enhancers (ESEs) [25]. ESE finder 3.0 predicted several high-confidence ESEs within CCL5 E2 (Fig. 7 C), suggesting that SRSF7 may inhibit CCL5 E2 skipping and promote CCL5 expression. RIP assays confirmed SRSF7 binding to CCL5 pre-mRNA (Fig. 7D). Using primers spanning E2 and E2-skipping events, PCR and gel electrophoresis revealed that SRSF7 knockdown increased CCL5 E2 skipping and decreased the proportion of mature mRNA containing E2 (Fig. 7E). Knockdown of SRSF7 in T24 and UMUC3 cells (Figure S7) reduced CCL5 mRNA and protein expression (Fig. 7F–I). Rescue experiments showed that HDAC7 knockdown promotes CCL5 expression, which is suppressed again if SRSF7 is also knocked down in T24 and UMUC3 cells (Fig. 7J–K, Figure S8).

### Virtual screening identifies pinocembrin as a specific inhibitor of HDAC7

**Fig. 8:**
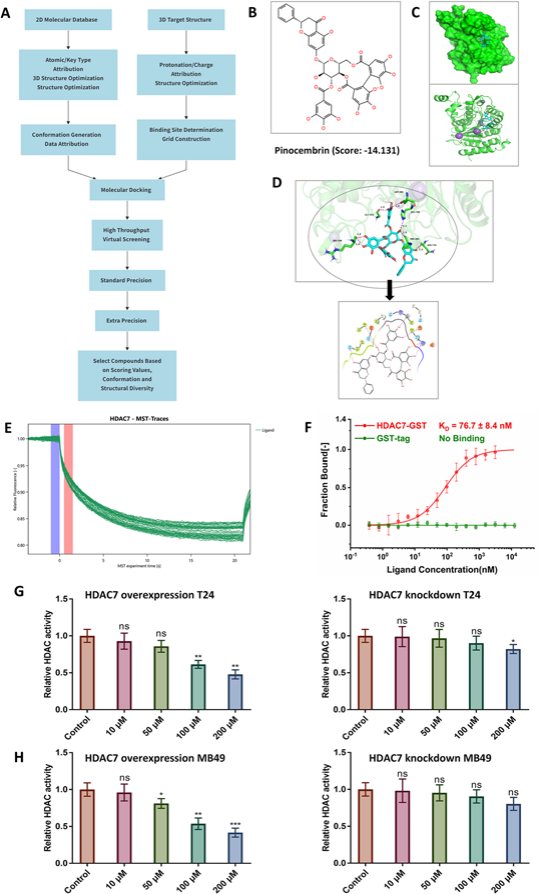
Virtual screening identifies Pinocembrin as a specific HDAC7 inhibitor.** A** Workflow of virtual screening for HDAC7 inhibitors. **B** Chemical structure of Pinocembrin and its predicted binding score with HDAC7. **C** Schematic diagrams showing Pinocembrin binding to HDAC7 in cartoon and surface representations. **D** Three-dimensional (3D) and two-dimensional (2D) views of Pinocembrin binding to HDAC7. In the 3D diagram, the C backbone of HDAC7 is green, N atoms blue, O atoms magenta, H atoms white, and Pinocembrin sky blue. Purple dashed lines indicate hydrogen bond lengths, with longer lines representing weaker interactions. E–F. MST experiments evaluate the binding affinity of Pinocembrin to HDAC7. G–H. T24 and MB49 cells were treated with increasing concentrations of Pinocembrin (10, 50, 100, 200 µM); HDAC activity was assessed in cells with different HDAC7 expression levels using an HDAC Activity Assay Kit (*P < 0.05, **P < 0.01, ***P < 0.001). Data are mean ± SD, *n* = 3

Most available HDAC inhibitors are non-selective, limiting their efficacy against solid tumors and increasing the risk of resistance and adverse effects [22]. Therefore, highly selective HDAC inhibitors are needed to improve therapeutic efficacy and minimize off-target toxicity. Here, virtual screening and molecular docking identified Pinocembrin as having the highest docking score for HDAC7 (Fig. 8A–D, Table S4−5). Pinocembrin, a flavonoid from propolis, has demonstrated antitumor effects in various malignancies [42], but its role in modulating immunotherapy sensitivity remains unclear. MST analysis demonstrated a dissociation constant (KD) of 76.7 nM between Pinocembrin and HDAC7 (Fig. 8E–F). Treatment of HDAC7-overexpressing and HDAC7 knockdown T24 cells with Pinocembrin showed that Pinocembrin significantly inhibited HDAC activity in HDAC7-overexpressing cells, but had limited effects in HDAC7 knockdown cells (Fig. 8G); similar results were observed in MB49 cells (Fig. 8H). These findings indicate that Pinocembrin is a potential specific inhibitor of HDAC7. To determine whether pinocembrin modulates the SRSF7–CCL5 axis, T24 and UMUC3 cells were treated with increasing concentrations of pinocembrin. The compound significantly elevated both the acetylation level and total expression of SRSF7, accompanied by a parallel up-regulation of CCL5 expression, indicating that pinocembrin activates the SRSF7–CCL5 pathway in BCa cells (Figure S9). To examine whether pinocembrin broadly inhibits other HDAC family members, T24 cells were transiently transfected with individual overexpression plasmids for HDACs 1–6 and 8–11, followed by treatment with 200 µM pinocembrin. HDAC activity analyses revealed no significant reduction in all groups, indicating that pinocembrin does not exert a general suppressive effect on HDAC activity (Figure S10A). In order to test the effect of Pinocembrin on the acetylation of known non-histone substrates of other HDACs, we treated T24 cells with 100 and 200 µM pinocembrin, then we detected the expression of LSD1, CDK1, MSH6, and α-Tubulin through Western blot. The results showed that pinocembrin did not alter the expression of LSD1, CDK1, MSH6 or α-tubulin, suggesting that it likewise exerts no appreciable effect on their acetylation status (Figure S10B).

### *In vivo* experiments confirm that pinocembrin enhances BCa immunotherapy sensitivity

**Fig. 9:**
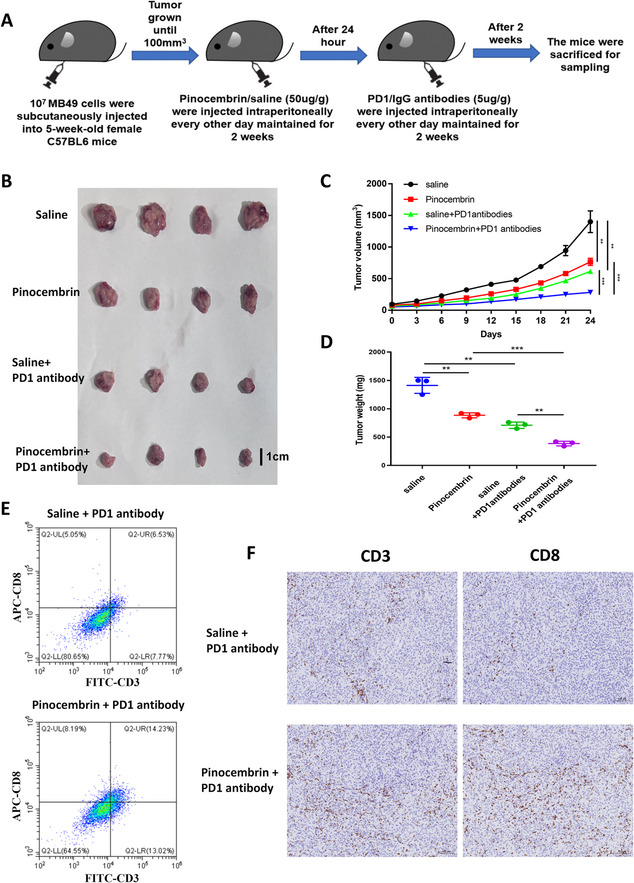
In vivo experiments demonstrate that Pinocembrin sensitizes BCa to immunotherapy. **A** Schematic of C57BL/6 tumor-bearing mice treated with Pinocembrin and immunotherapy. **B** C57BL/6 mice with subcutaneous xenografts received regular Pinocembrin and anti-PD1 treatment. Tumors were harvested after observation and measurement. **C** Tumor volume measured every 3 days in C57BL/6 mice (**P < 0.01, ***P < 0.001). **D** Tumor weight in C57BL/6 mice assessed after dissection (**P < 0.01, ***P < 0.001). **E** Flow cytometry analysis of CD3 and CD8 positivity in tumor tissues. **F** IHC detection of CD3 and CD8 positivity in tumor tissues. Data are mean ± SD, *n* = 4

To assess the value of Pinocembrin in combination with immunotherapy for BCa, a C57BL/6 mouse model bearing MB49 tumors was treated with Pinocembrin and anti-PD1 (Fig. 9A). Pinocembrin inhibited BCa proliferation in vivo, and its combination with anti-PD1 demonstrated significantly greater therapeutic efficacy than anti-PD1 alone (Fig. 9B–D). Flow cytometry and immunohistochemistry confirmed that CD8 + T cell tumor infiltration was significantly higher in the combination group than in the single-agent group (Fig. 9E–F). Collectively, these results demonstrate that the HDAC7 inhibitor Pinocembrin can enhance BCa immunotherapy sensitivity.

## Discussion

In this study, HDAC7 was identified as a key modulator of immunotherapy sensitivity in BCa through retrospective analysis of the IMvigor210 clinical trial cohort and follow-up data from locally advanced BCa patients treated with adjuvant immunotherapy at our center. We confirmed that HDAC7 is highly expressed in BCa and is associated with poor prognosis. Functional experiments demonstrated that HDAC7 suppresses CD8 + T cell chemotaxis within the BCa TME, thereby diminishing the efficacy of immunotherapy. Mechanistically, HDAC7 deacetylates lysine 24 of SRSF7, promoting BTRC-mediated ubiquitination and degradation of SRSF7, which in turn inhibits the maturation of CCL5 pre-mRNA and reduces CCL5 expression. Therefore, CD8 + T cell infiltration is impeded. Virtual screening further identified Pinocembrin as a specific inhibitor of HDAC7, and in vivo studies showed that Pinocembrin enhances the immunotherapy sensitivity of BCa. Collectively, these findings highlight the potential of HDAC7 as a novel biomarker for predicting and monitoring immunotherapy sensitivity in BCa, and demonstrate that Pinocembrin may be a promising agent to overcome BCa immunotherapy resistance.

BCa remains a significant global health challenge, and ICI-based immunotherapy has emerged as a promising treatment approach [43]. However, the overall response rate to ICIs remains low, with considerable interindividual variability in treatment results [44]. These limitations highlight the need to elucidate mechanisms underlying immunotherapy resistance and to develop strategies that improve treatment efficacy. Mechanisms shown to alter the immune phenotype and enhance immunotherapy efficacy include induction of localized tumor inflammation, reversal of the immunosuppressive TME, remodeling of tumor vasculature, targeting of tumor-intrinsic signaling pathways, and augmentation of tumor-specific T cell responses [45]. CD8 + T cells are pivotal effectors in antitumor immunity, and their abundance and functional status directly influence immunotherapy sensitivity [46]. Classification of tumor immune phenotypes—immune-inflamed, immune-excluded, and immune-desert—by the degree of intratumoral lymphocyte infiltration provides insight into immunotherapy response [47]. Tumors with abundant TILs (immune-inflamed) respond best to immunotherapy, whereas immune-excluded and immune-desert tumors, which lack substantial T cell infiltration, are less responsive. These observations highlight the importance of strategies that enhance CD8 + T cell infiltration to optimize immunotherapy efficacy. For instance, B7-H4 and EZH2 have been shown to limit CD8 + T cell infiltration in glioma and esophageal squamous cell carcinoma, respectively, providing new potential therapeutic targets [48, 49].

HDACs regulate gene expression by deacetylating histone and non-histone proteins [50–52]. However, the function of individual HDAC subtypes is heterogeneous across tumor types. For example, in NSCLC, HDAC1 facilitates disease progression via EGFR/AKT signaling and can promote resistance to EGFR tyrosine kinase inhibitors through downregulation of miR-33a [53, 54]. Conversely, HDAC1 acts as a tumor suppressor in ALK-positive anaplastic large cell lymphoma [55]. HDAC2 has been implicated in the malignant phenotype of hepatocellular carcinoma via LAPTM4B activation and in cisplatin resistance in triple-negative breast cancer by modulating m6A-mediated DNA damage repair [56, 57]. These findings emphasize the need to define HDAC subtype-specific functions within each cancer type. Only by targeting relevant HDAC subtypes can resistance to BCa immunotherapy be effectively addressed.

A range of HDACis have been developed, including vorinostat, romidepsin, and belinostat, which are approved mainly for hematological malignancies [21, 58]. However, their application in solid tumors, including BCa, is limited by toxicity and insufficient specificity [59]. Recent studies indicate that subtype-specific HDAC inhibitors have superior therapeutic potential. For example, the highly selective HDAC11 inhibitor B6 has demonstrated favorable pharmacokinetics in the treatment of metabolic dysfunction-associated fatty liver disease [60], and selective inhibition of HDAC6 by N-acylhydrazone can suppress hepatocellular carcinoma progression [61]. Thus, identification of HDAC subtype-selective inhibitors is essential to minimize off-target toxicity and improve therapeutic efficacy in BCa.

This study opens several directions for future research. The role of HDAC7 in different BCa subtypes and its interactions with other immune checkpoint pathways warrant further investigation. Large, multicenter clinical trials are required to validate HDAC7 as a predictive and monitoring biomarker for BCa immunotherapy. Additionally, preclinical studies are needed to evaluate the pharmacokinetics, toxicology, and efficacy of Pinocembrin across various BCa models.

## Conclusion

**Fig. 10:**
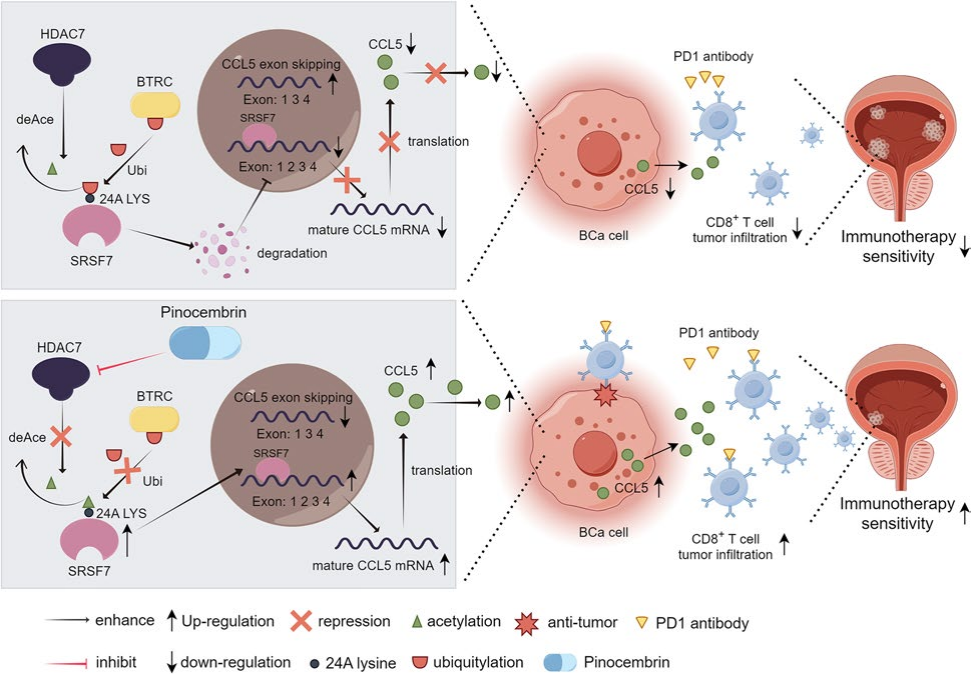
Schematic model of the Pinocembrin/HDAC7/BTRC/SRSF7/CCL5 regulatory and functional network

This study demonstrates that HDAC7 promotes BTRC-mediated ubiquitination and degradation of SRSF7 via non-histone deacetylation, resulting in impaired CCL5 pre-mRNA maturation and reduced CD8 + T cell chemotaxis to tumors, thereby mediating immunotherapy resistance in BCa. The HDAC7-specific inhibitor Pinocembrin enhances BCa sensitivity to immunotherapy (Fig. 10). Therefore, HDAC7 represents a promising biomarker and therapeutic target for predicting immunotherapy sensitivity in BCa, and pharmacological inhibition of HDAC7 may offer a novel therapeutic strategy for BCa.

## Supplementary Information

Supplementary Material 1: Table S1: All siRNAs used in this research.

Supplementary Material 2: Table S2: All PCR primers used in this research

Supplementary Material 3: Table S3: Summary of relations between HDAC7 and Cancer Immunotherapy Response Data

Supplementary Material 4: Table S4: Detailed Information of the Top 200 Compounds Predicted from the HY-L001V Database

Supplementary Material 5: Table S5: Detailed Information of the Top 200 Compounds Predicted from the HY-L032V Database

Supplementary Material 6: Figure S1. Retrospective analysis of the IMvigor210 cohort reveals no significant correlation between other HDACs and BCa immunotherapy response.A–I. Correlation analysis of HDACs (HDAC2–6 and HDAC8–11) with BCa immunotherapy response using IMvigor210 cohort data

Supplementary Material 7: Figure S2. Expression of HDAC7 across TCGA pan-cancer types. A. HDAC7 expression in various TCGA cancer types compared with normal tissues (**P < 0.01, ***P < 0.001). B. HDAC7 expression in tumor vs. normal tissues across all cancer types (***P < 0.001). C. HDAC7 expression in 31 different tumor types

Supplementary Material 8: Figure S3. HDAC7 promotes BCa cell proliferation. A–B. CCK8 assays in HDAC7 knockdown T24 and HDAC7-overexpressing UMUC3 cells (*P < 0.05,**P < 0.01). C–F. Colony formation assays in HDAC7 knockdown T24 and HDAC7-overexpressing UMUC3 cells (*P < 0.05,**P < 0.01). Data are mean ± SD, *n* = 3.

Supplementary Material 9: Figure S4. CCL5 rescues HDAC7-mediated suppression of CD8+ T cell infiltration A. ELISA validation of HDAC7 knockdown and CCL5 siRNA transfection efficiency in T24 cells (**P < 0.01, ***P < 0.001). B. Co-culture assays show that CCL5 knockdown reverses the increase in CD8+ T cell chemotaxis caused by HDAC7 knockdown in T24 cells (**P < 0.01). C. ELISA validation in UMUC3 cells (*P < 0.05, **P < 0.01). D. Co-culture assays confirm reversal of CD8+ T cell chemotaxis promotion by CCL5 knockdown in UMUC3 cells (*P < 0.05). Data are mean ± SD, *n *= 3.

Supplementary Material 10: Figure S5. HDAC7 does not affect SRSF7 mRNA expression. A. qRT-PCR for SRSF7 mRNA in T24 cells with HDAC7 knockdown. B. qRT-PCR for SRSF7 mRNA in UMUC3 cells with HDAC7 overexpression. Data are mean ± SD, *n* = 3. Figure S6. HDAC7 knockdown inhibits SRSF7 ubiquitination in UMUC3 cells. A. CO-IP showing that HDAC7 knockdown inhibits SRSF7 ubiquitination. Data are mean ± SD, *n* = 3.

Supplementary Material 11: Figure S6. HDAC7 knockdown inhibits SRSF7 ubiquitination in UMUC3 cells. A. CO-IP showing that HDAC7 knockdown inhibits SRSF7 ubiquitination. Data are mean± SD, *n* = 3.

Supplementary Material 12: Figure S7. Efficiency of siRNA-mediated SRSF7 knockdown in BCa. A–B. Transfection efficiency of SRSF7 siRNAs in T24 and UMUC3 cells (**P< 0.01, ***P < 0.001). Data are mean ± SD, *n* = 3.

Supplementary Material 13: Figure S8. SRSF7 rescued the decreased CCL5. A-B. qRT-PCR and ELISA-based rescue experiments demonstrated that HDAC7 knockdown increases CCL5 expression, which is repressed when SRSF7 is simultaneously knocked down in UMUC3 cells (*P < 0.05, **P < 0.01). Data are mean± SD, *n* = 3.

Supplementary Material 14: Figure S9. Pinocembrin promoted SRSF7-CCL5 expression. A-B. T24 and UMUC3 cells were treated with increasing concentrations of Pinocembrin (10, 50, 100, 200 μM). Western blot results showed Pinocembrin could promoted the acetylation and expression of SRSF7. C-D. T24 and UMUC3 cells were treated with increasing concentrations of Pinocembrin (10, 50, 100, 200 μM). ELISA results showed Pinocembrin could promoted the expression of CCL5 (*P < 0.05, **P < 0.01, ***P < 0.001). Data are mean ± SD, *n* = 3.

Supplementary Material 15: Figure S10. Pinocembrin does not exert a general suppressive effect on HDAC activity. A. T24 cells were treated with 200 μM concentration of Pinocembrin, HDAC activity was assessed in cells with different HDACs (HDAC1-6 and 8-11) expression levels using an HDAC Activity Assay Kit. B. The effect of Pinocembrin on the expression of known non-histone substrates of other HDACs, such as LSD1, CDK1, MSH6, and α-Tubulin, were studied through Western blot. Data are mean ± SD, *n*= 3.

Supplementary Material 16

## Data Availability

The datasets supporting the conclusions of this article are included within the article and its additional files.

## Abbreviations

BCa: Bladder cancer
TME: Tumour microenvironment
NMIBC: Non-muscle invasive bladder cancer
MIBC: Muscle invasive bladder cancer
ICIs: immune checkpoint inhibitors
HDAC: histone deacetylase
RBPs: RNA binding proteins
SRSF: Serine and arginine rich splicing factor
BTRC: Beta-Transducin Repeat Containing E3 Ubiquitin Protein Ligase
CCL5: C-C Motif chemokine ligand 5
Pinocembrin: Pinocembrin 7-O-[3’’-O-galloyl-4’’,6’’-hexahydroxydiphenoyl] β-D-glucoside
RIP: RNA immunoprecipitation
CO-IP: Co-Immunoprecipitation
ELISA: Enzyme-linked immunosorbent assay
CHIP: Chromatin immunoprecipitation
PBMCs: Peripheral blood mononuclear cells
HuNOG mice: Human induced NOD-scid IL2Rgnull mice
IHC: Immunohistochemistry
MST: MicroScale Thermophoresis
