# Supplementary material for: Histone deacetylase HDAC7 restricts CD8 + T cell tumor infiltration and limits immunotherapy sensitivity in bladder cancer: reversal by pinocembrin
Source: J Exp Clin Cancer Res. 2025 Dec 24;44:324. doi: 10.1186/s13046-025-03585-3 (PMC12729082; doi:10.1186/s13046-025-03585-3)
Supplement: Supplementary file 1 — Supplementary Material 1: Table S1: All siRNAs used in this research. [file 13046_2025_3585_MOESM1_ESM.doc]

**Table S1:** All siRNAs used in this research.

| **Gene/circRNA** | **Sequences (5’-3’)** |
| --- | --- |
| HDAC7 siRNA-1  HDAC7 siRNA-2 | AAGUAGUUGGAACCAGAGAA  ACUGACCUCGCCUUCAAAGTT |
| SRSF7 siRNA-1  SRSF7 siRNA-2 | AGGAGAGUUAGAAAGGGCUTT  GCAUCUCCUCGACGAUCAATT |
| BTRC siRNA-1  BTRC siRNA-2 | GUGGAUUCUCAGACAUGAUACUCTC  AUUGUCAGUAGUUCACAUGAUGACA |
| CCL5 siRNA-1  CCL5 siRNA-2 | CCTCGCTGTCATCCTCATT  GAGAAGAAGTGGGTTCAAGAA |
