# Supplementary material for: Histone deacetylase HDAC7 restricts CD8 + T cell tumor infiltration and limits immunotherapy sensitivity in bladder cancer: reversal by pinocembrin
Source: J Exp Clin Cancer Res. 2025 Dec 24;44:324. doi: 10.1186/s13046-025-03585-3 (PMC12729082; doi:10.1186/s13046-025-03585-3)
Supplement: Supplementary file 2 — Supplementary Material 2: Table S2: All PCR primers used in this research [file 13046_2025_3585_MOESM2_ESM.doc]

**Table S2:** All PCR primers used in this research.

| **Primers and probes** |  | **sequence** |
| --- | --- | --- |
| HDAC7  HDAC1 | Forward  Reverse  Forward  Reverse | 5’-GGCGGCCCTAGAAAGAACAG-3’  5’-CTTGGGCTTATAGCGCAGCTT-3’  5’-CCGCATGACTCATAATTTGCTG-3’  5’-ATTGGCTTTGTGAGGGCGATA-3’ |
| SRSF7  CCL5  BTRC  β-Actin | Forward  Reverse  Forward  Reverse  Forward  Reverse  Forward  Reverse | 5’-CGGTACGGAGGAGAAACCAAG-3’  5’-AGCCACAAATCACCTTTCCATC-3’  5’-GAGCGGGTGGGGTAGGATAGTGAGG-3’  5’-CCACACCCTGCTGCTTTGCCTACAT-3’  5’-TGCCCAAGCAACGGAAACT-3’  5’-GCCCATGTTGGTAATGACACA-3’  5’-CTCCATCCTGGCCTCGCTGT-3’  5’-GCTGTCACCTTCACCGTTCC-3’ |
