# Supplementary material for: Histone deacetylase HDAC7 restricts CD8 + T cell tumor infiltration and limits immunotherapy sensitivity in bladder cancer: reversal by pinocembrin
Source: J Exp Clin Cancer Res. 2025 Dec 24;44:324. doi: 10.1186/s13046-025-03585-3 (PMC12729082; doi:10.1186/s13046-025-03585-3)
Supplement: Supplementary file 3 — Supplementary Material 3: Table S3: Summary of relations between HDAC7 and Cancer Immunotherapy Response Data [file 13046_2025_3585_MOESM3_ESM.doc]

**Table S3:** **Summary of Cancer Immunotherapy Response Data**

| **No** | **PMID** | **Cancer type** | **Group** | **Drug** | **# Res** | **# NRes** | **Log2 (Fold Change)** | **P value** |  |
| --- | --- | --- | --- | --- | --- | --- | --- | --- | --- |
| 1 | 26997480 | Melanoma | all | Anti-PD-1 (pembrolizumab and nivolumab) | 14 | 12 | -0.036 | 0.903 |  |
| 2 | 26997480 | Melanoma | MAPKi | Anti-PD-1 (pembrolizumab and nivolumab) | 6 | 5 | -0.288 | 0.882 |  |
| 3 | 26997480 | Melanoma | non-MAPKi | Anti-PD-1 (pembrolizumab and nivolumab) | 8 | 7 | 0.154 | 0.908 |  |
| 4 | 28552987 | Urothelial cancer | all | Anti-PD-L1 (atezolizumab) | 9 | 16 | 0.082 | 0.805 |  |
| 5 | 28552987 | Urothelial cancer | smoking | Anti-PD-L1 (atezolizumab) | 5 | 9 | 0.13 | 0.959 |  |
| 6 | 28552987 | Urothelial cancer | non-smoking | Anti-PD-L1 (atezolizumab) | 4 | 7 | 0.025 | 0.994 |  |
| 7 | 29033130 | Melanoma | all | Anti-PD-1 (nivolumab) | 26 | 23 | 0.191 | 0.618 |  |
| 8 | 29033130 | Melanoma | NIV3-PROG | Anti-PD-1 (nivolumab) | 15 | 11 | 0.25 | 0.872 |  |
| 9 | 29033130 | Melanoma | NIV3-NAIVE | Anti-PD-1 (nivolumab) | 11 | 12 | 0.151 | 0.929 |  |
| 10 | 29301960 | Clear cell renal cell carcinoma (ccRCC) | all | Anti-PD-1 (nivolumab) | 4 | 8 | 0.672 | 0.691 |  |
| 11 | 29301960 | Clear cell renal cell carcinoma (ccRCC) | VEGFRi | Anti-PD-1 (nivolumab) | 2 | 0 | 0 | 1 |  |
| 12 | 29301960 | Clear cell renal cell carcinoma (ccRCC) | non-VEGFRi | Anti-PD-1 (nivolumab) | 2 | 8 | 1.416 | 0.559 |  |
| 13 | 29443960 | Urothelial cancer | all | Anti-PD-L1 (atezolizumab) | 68 | 230 | -0.207 | 0.00026 |  |
|  | | | | | | | | | |
