# Supplementary figures and images for: Histone deacetylase HDAC7 restricts CD8 + T cell tumor infiltration and limits immunotherapy sensitivity in bladder cancer: reversal by pinocembrin
Source: J Exp Clin Cancer Res. 2025 Dec 24;44:324. doi: 10.1186/s13046-025-03585-3 (PMC12729082; doi:10.1186/s13046-025-03585-3)

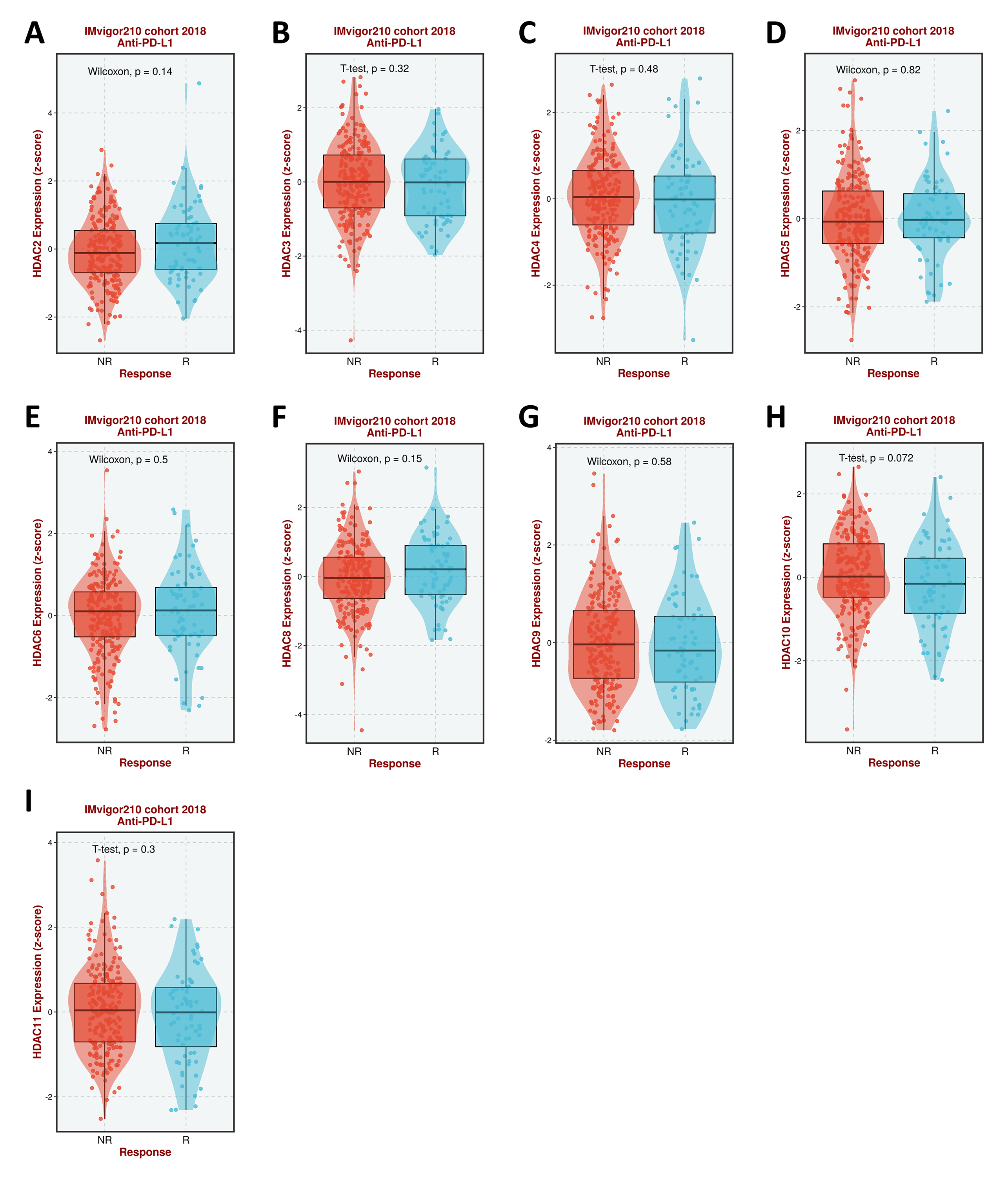

Supplement: Supplementary file 6 — Supplementary Material 6: Figure S1. Retrospective analysis of the IMvigor210 cohort reveals no significant correlation between other HDACs and BCa immunotherapy response.A–I. Correlation analysis of HDACs (HDAC2–6 and HDAC8–11) with BCa immunotherapy response using IMvigor210 cohort data [file 13046_2025_3585_MOESM6_ESM.tif]

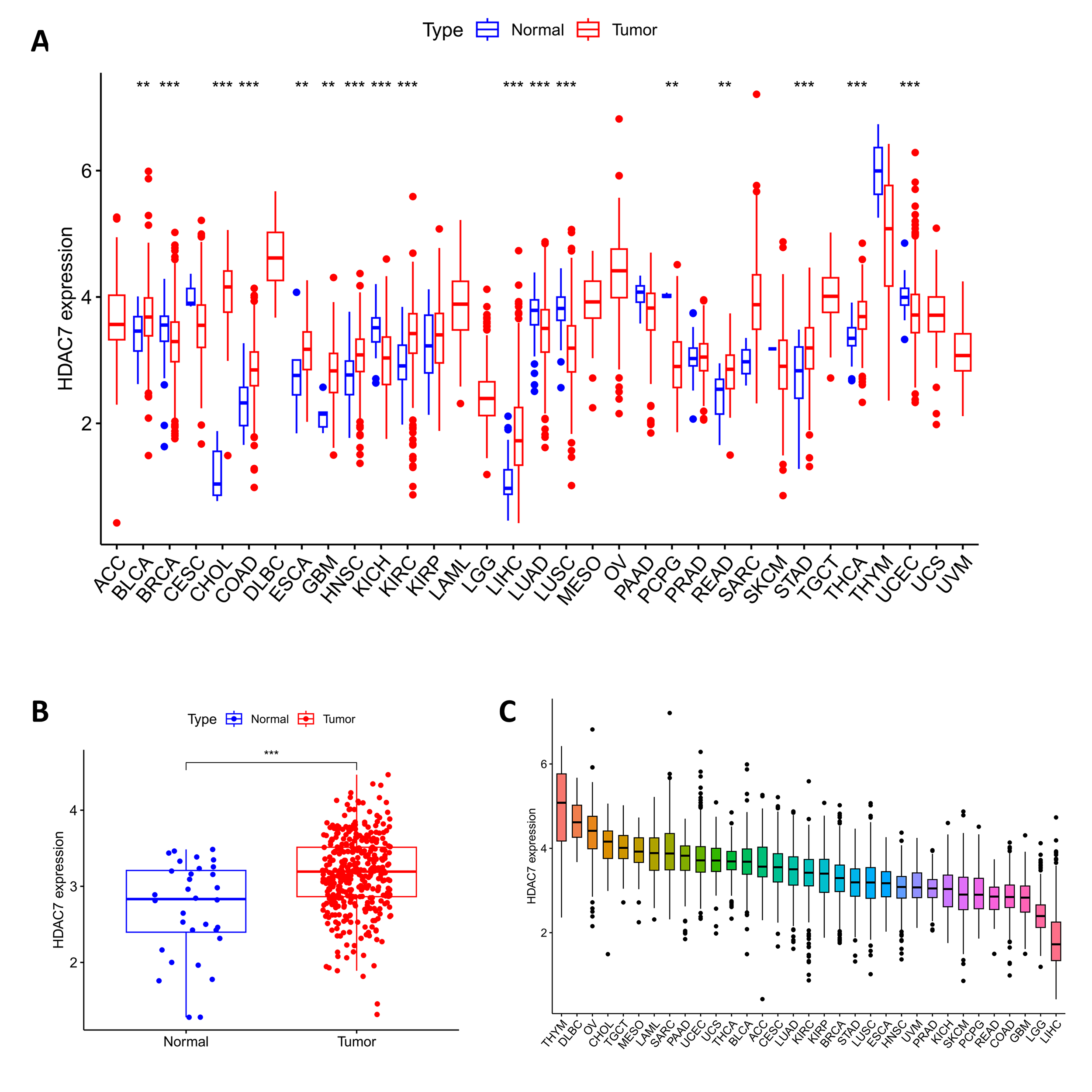

Supplement: Supplementary file 7 — Supplementary Material 7: Figure S2. Expression of HDAC7 across TCGA pan-cancer types. A. HDAC7 expression in various TCGA cancer types compared with normal tissues (**P < 0.01, ***P < 0.001). B. HDAC7 expression in tumor vs. normal tissues across all cancer types (***P < 0.001). C. HDAC7 expression in 31 different tumor types [file 13046_2025_3585_MOESM7_ESM.tif]

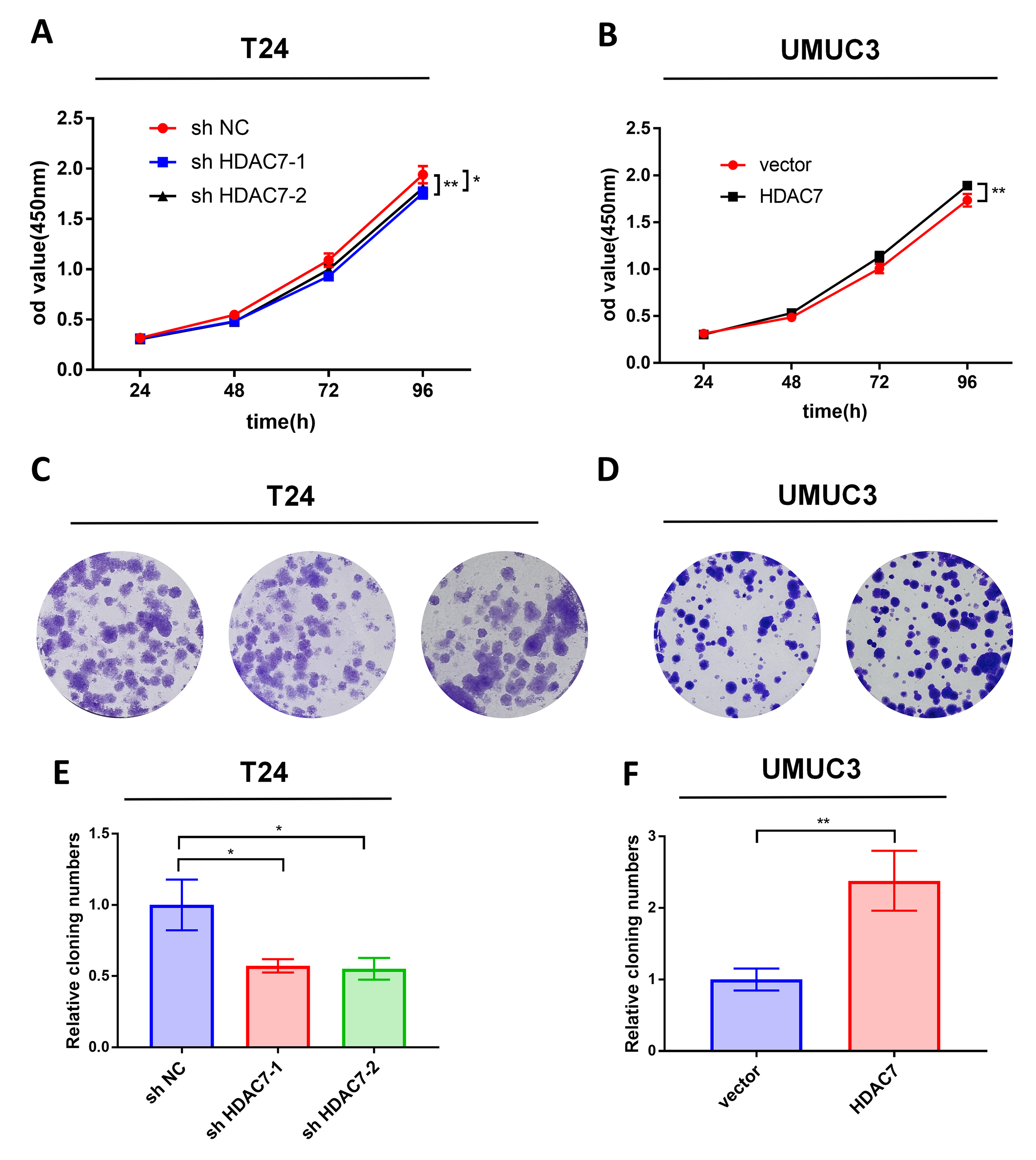

Supplement: Supplementary file 8 — Supplementary Material 8: Figure S3. HDAC7 promotes BCa cell proliferation. A–B. CCK8 assays in HDAC7 knockdown T24 and HDAC7-overexpressing UMUC3 cells (*P < 0.05,**P < 0.01). C–F. Colony formation assays in HDAC7 knockdown T24 and HDAC7-overexpressing UMUC3 cells (*P < 0.05,**P < 0.01). Data are mean ± SD, n = 3. [file 13046_2025_3585_MOESM8_ESM.tif]

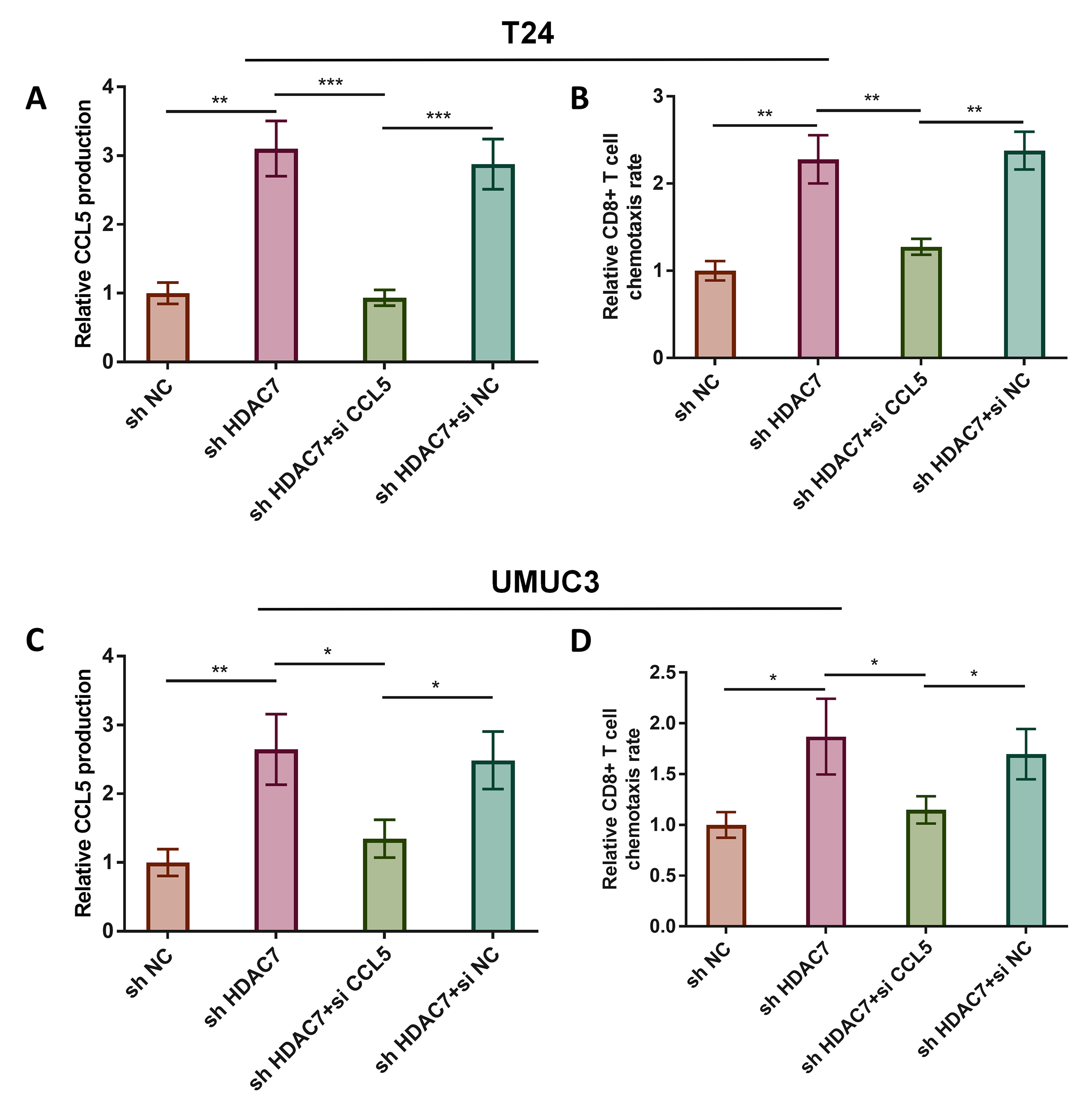

Supplement: Supplementary file 9 — Supplementary Material 9: Figure S4. CCL5 rescues HDAC7-mediated suppression of CD8+ T cell infiltration A. ELISA validation of HDAC7 knockdown and CCL5 siRNA transfection efficiency in T24 cells (**P < 0.01, ***P < 0.001). B. Co-culture assays show that CCL5 knockdown reverses the increase in CD8+ T cell chemotaxis caused by HDAC7 knockdown in T24 cells (**P < 0.01). C. ELISA validation in UMUC3 cells (*P < 0.05, **P < 0.01). D. Co-culture assays confirm reversal of CD8+ T cell chemotaxis promotion by CCL5 knockdown in UMUC3 cells (*P < 0.05). Data are mean ± SD, n = 3. [file 13046_2025_3585_MOESM9_ESM.tif]

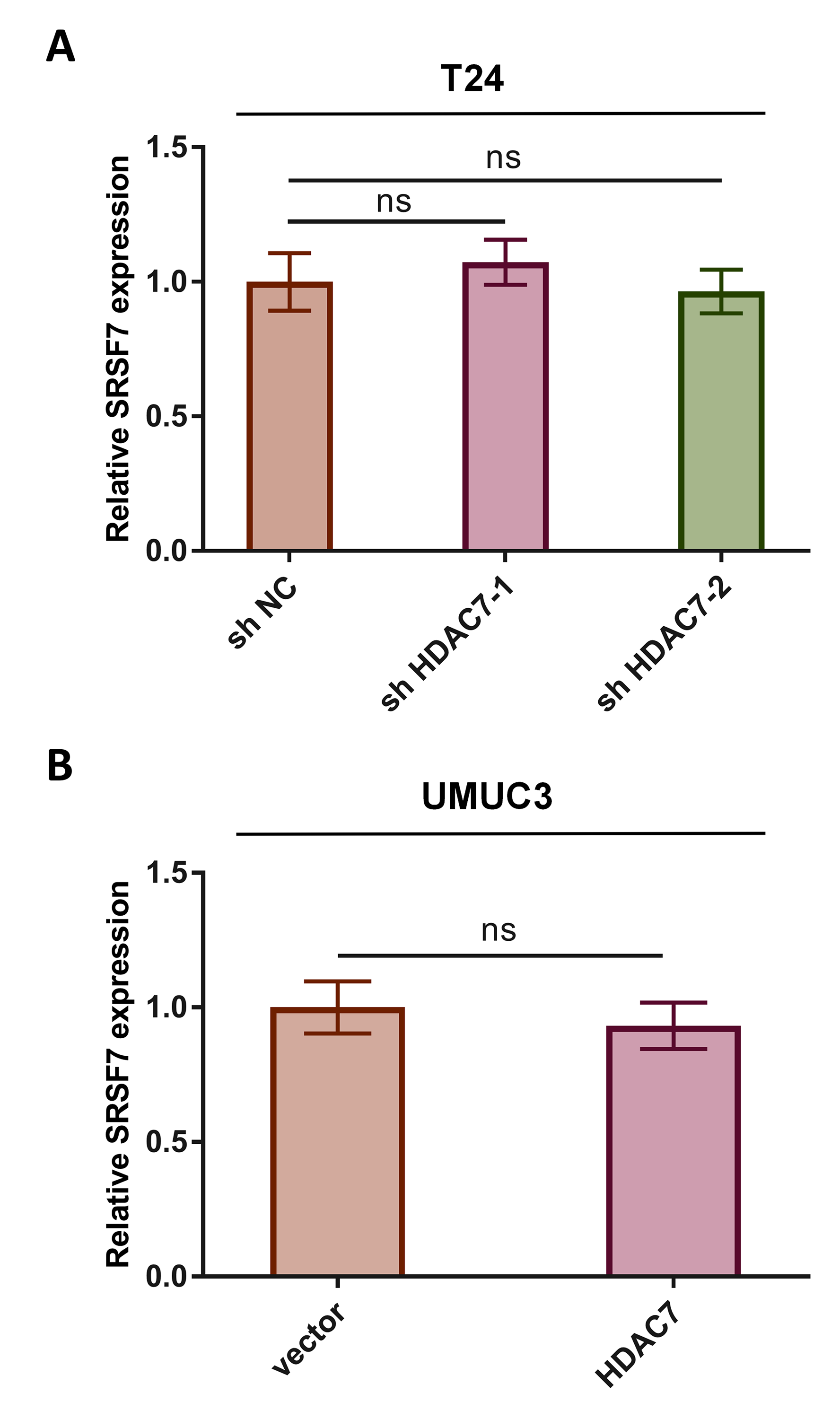

Supplement: Supplementary file 10 — Supplementary Material 10: Figure S5. HDAC7 does not affect SRSF7 mRNA expression. A. qRT-PCR for SRSF7 mRNA in T24 cells with HDAC7 knockdown. B. qRT-PCR for SRSF7 mRNA in UMUC3 cells with HDAC7 overexpression. Data are mean ± SD, n = 3. Figure S6. HDAC7 knockdown inhibits SRSF7 ubiquitination in UMUC3 cells. A. CO-IP showing that HDAC7 knockdown inhibits SRSF7 ubiquitination. Data are mean ± SD, n = 3. [file 13046_2025_3585_MOESM10_ESM.tif]

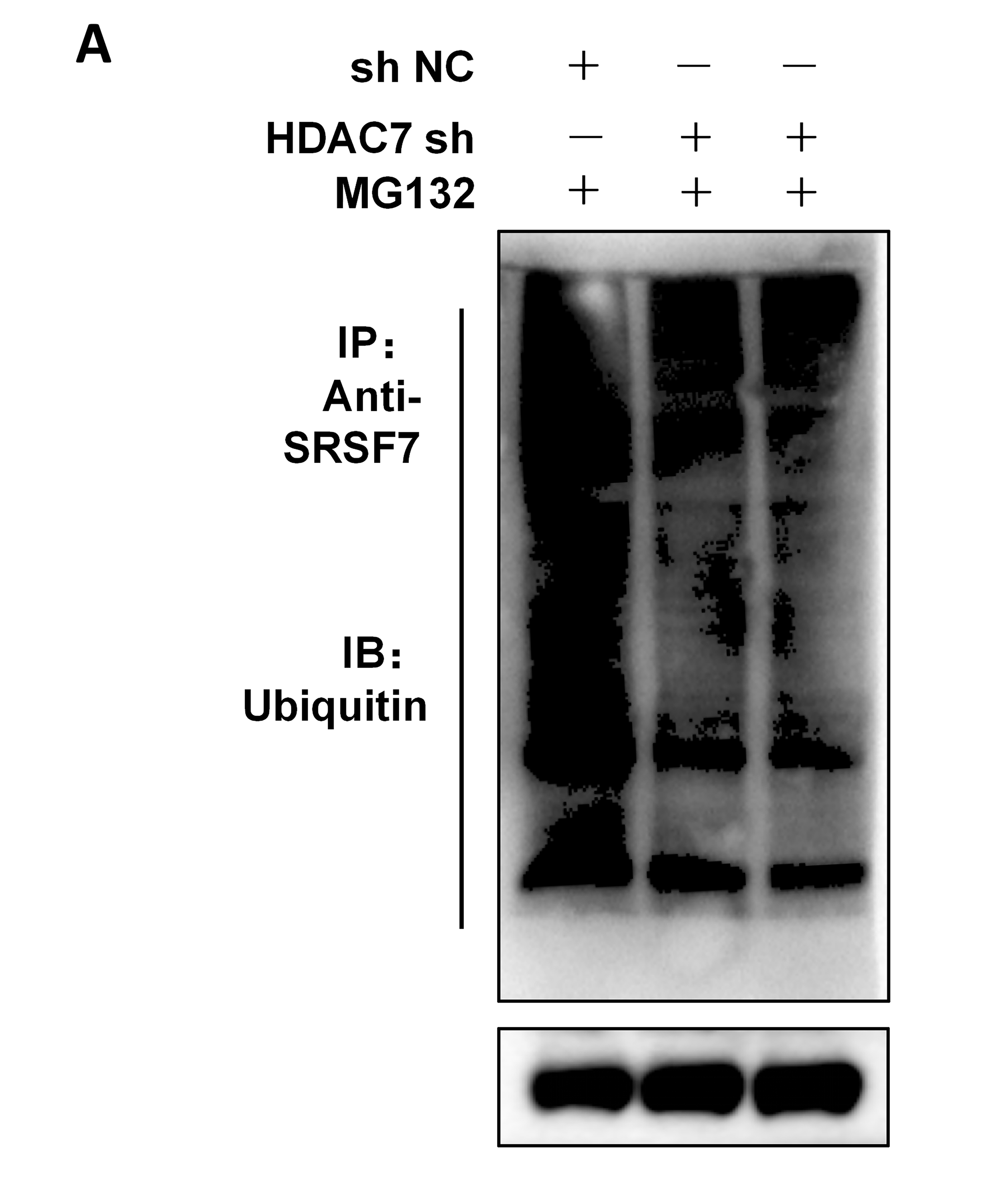

Supplement: Supplementary file 11 — Supplementary Material 11: Figure S6. HDAC7 knockdown inhibits SRSF7 ubiquitination in UMUC3 cells. A. CO-IP showing that HDAC7 knockdown inhibits SRSF7 ubiquitination. Data are mean± SD, n = 3. [file 13046_2025_3585_MOESM11_ESM.tif]

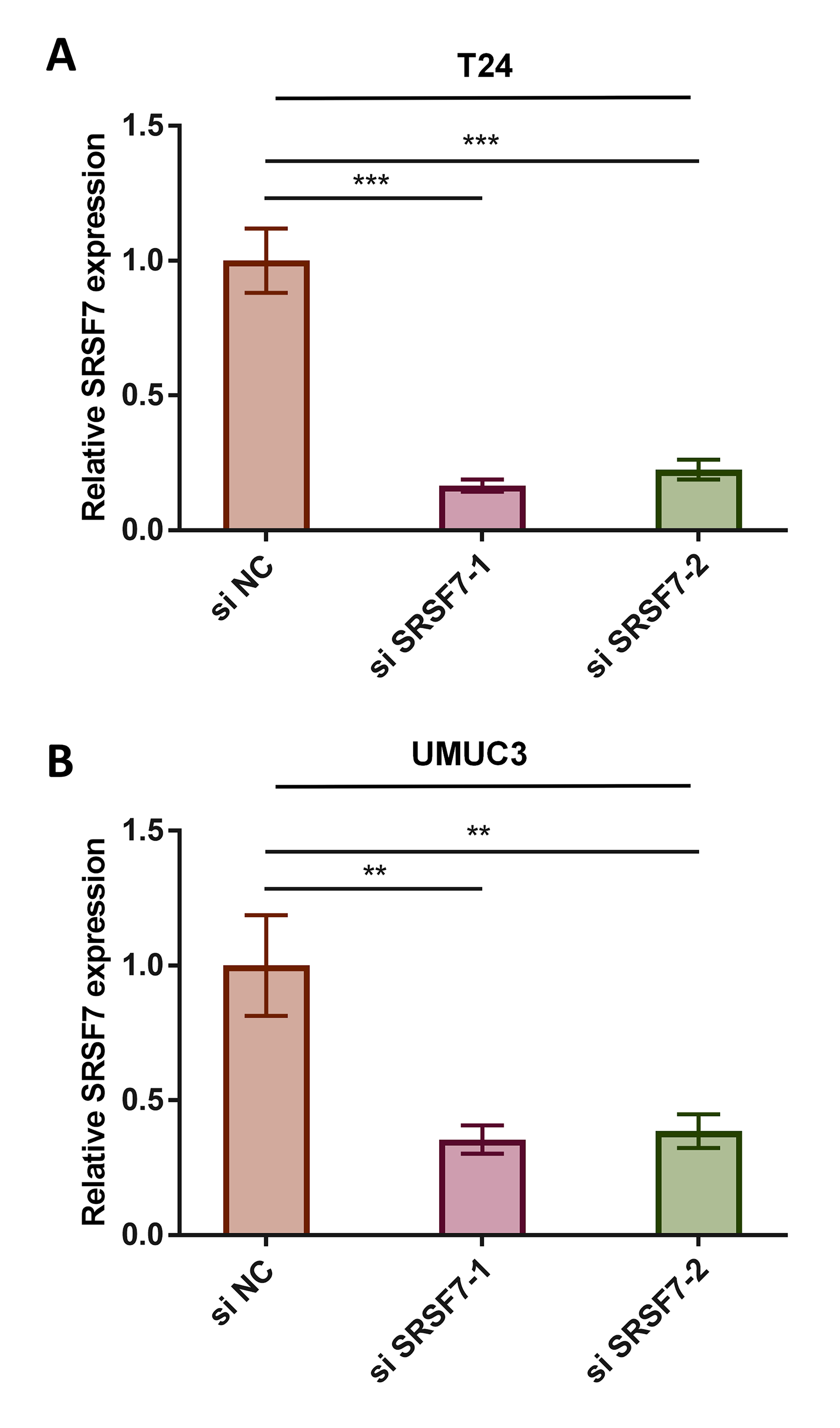

Supplement: Supplementary file 12 — Supplementary Material 12: Figure S7. Efficiency of siRNA-mediated SRSF7 knockdown in BCa. A–B. Transfection efficiency of SRSF7 siRNAs in T24 and UMUC3 cells (**P< 0.01, ***P < 0.001). Data are mean ± SD, n = 3. [file 13046_2025_3585_MOESM12_ESM.tif]

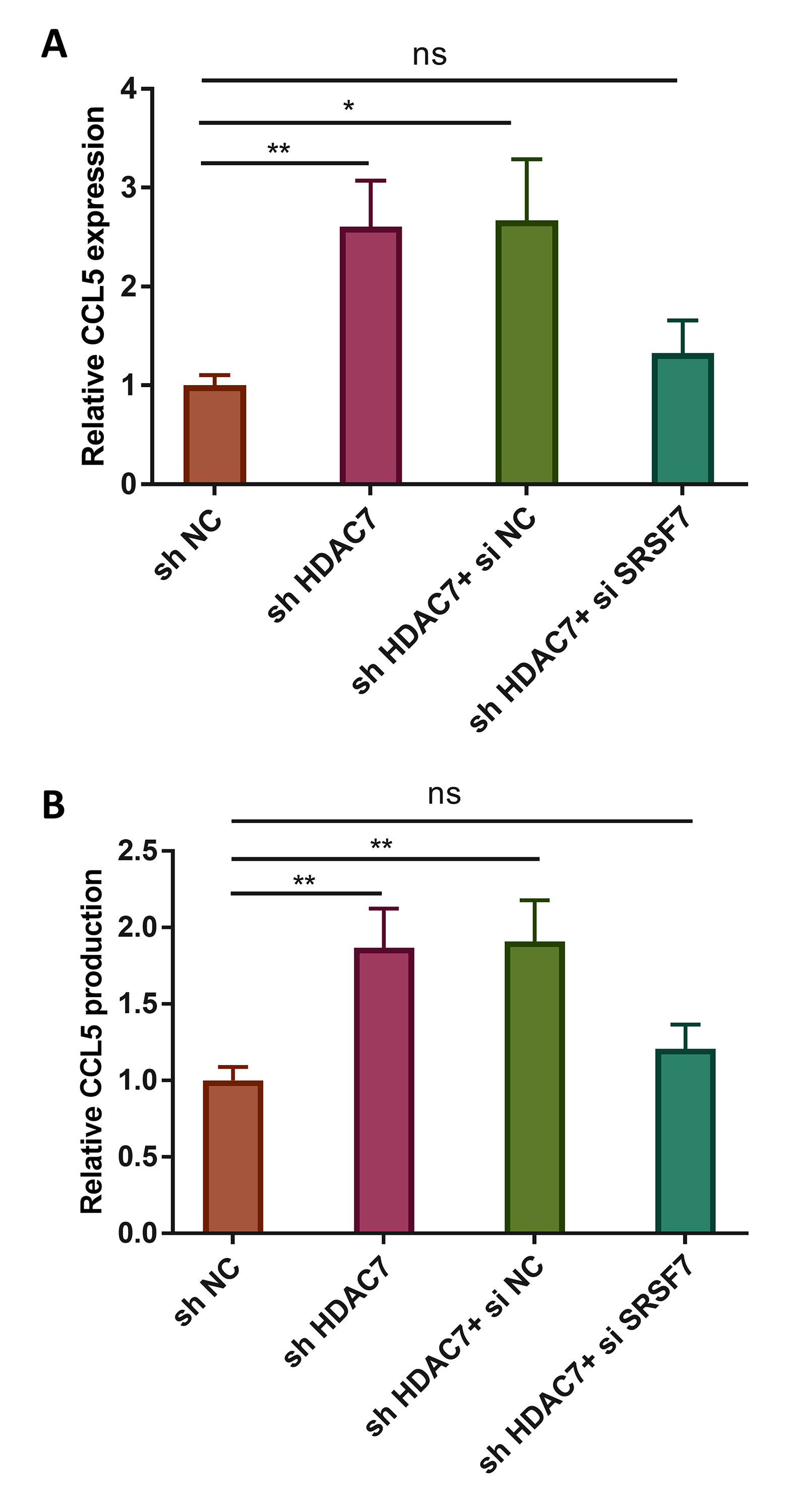

Supplement: Supplementary file 13 — Supplementary Material 13: Figure S8. SRSF7 rescued the decreased CCL5. A-B. qRT-PCR and ELISA-based rescue experiments demonstrated that HDAC7 knockdown increases CCL5 expression, which is repressed when SRSF7 is simultaneously knocked down in UMUC3 cells (*P < 0.05, **P < 0.01). Data are mean± SD, n = 3. [file 13046_2025_3585_MOESM13_ESM.tif]

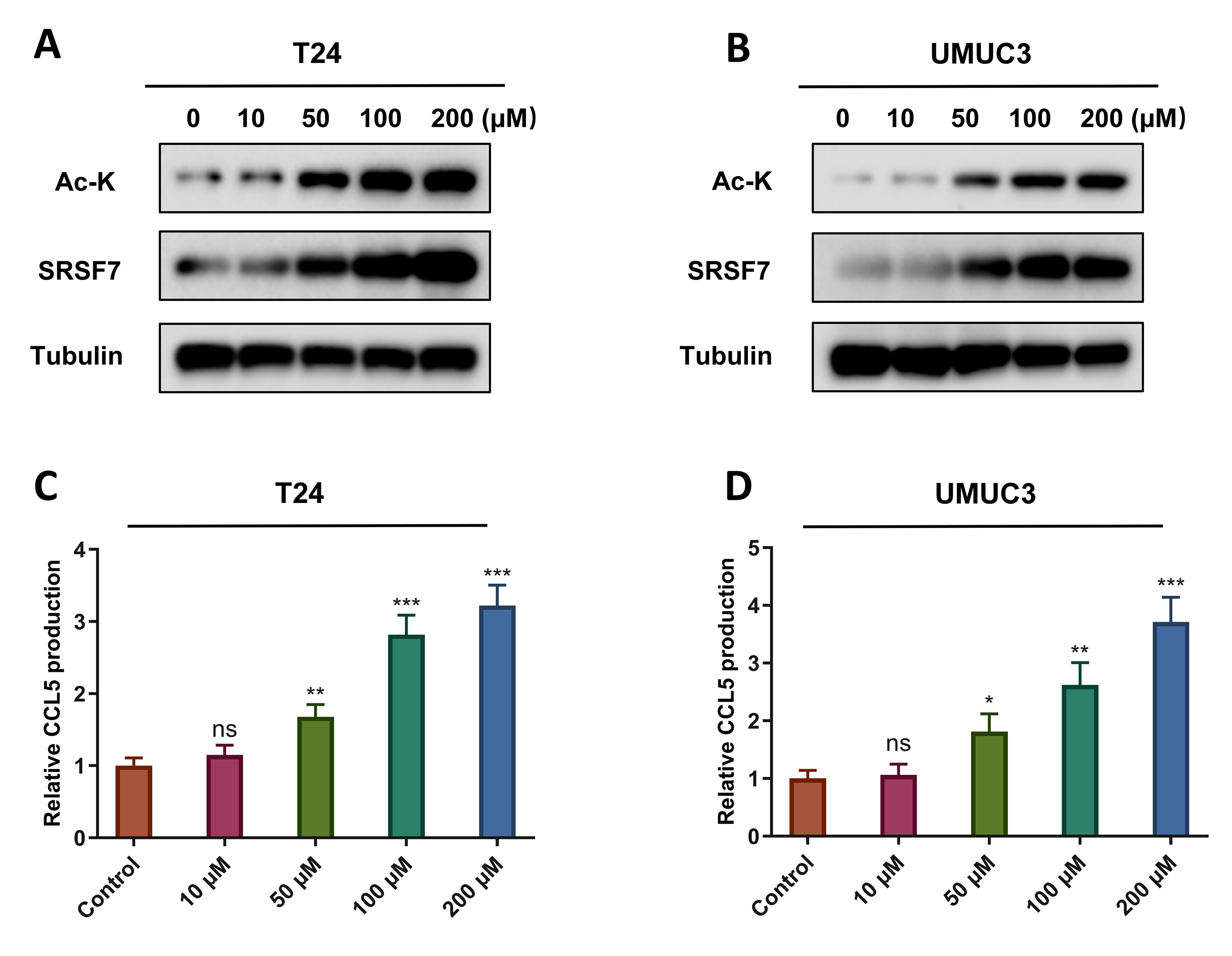

Supplement: Supplementary file 14 — Supplementary Material 14: Figure S9. Pinocembrin promoted SRSF7-CCL5 expression. A-B. T24 and UMUC3 cells were treated with increasing concentrations of Pinocembrin (10, 50, 100, 200 μM). Western blot results showed Pinocembrin could promoted the acetylation and expression of SRSF7. C-D. T24 and UMUC3 cells were treated with increasing concentrations of Pinocembrin (10, 50, 100, 200 μM). ELISA results showed Pinocembrin could promoted the expression of CCL5 (*P < 0.05, **P < 0.01, ***P < 0.001). Data are mean ± SD, n = 3. [file 13046_2025_3585_MOESM14_ESM.tif]

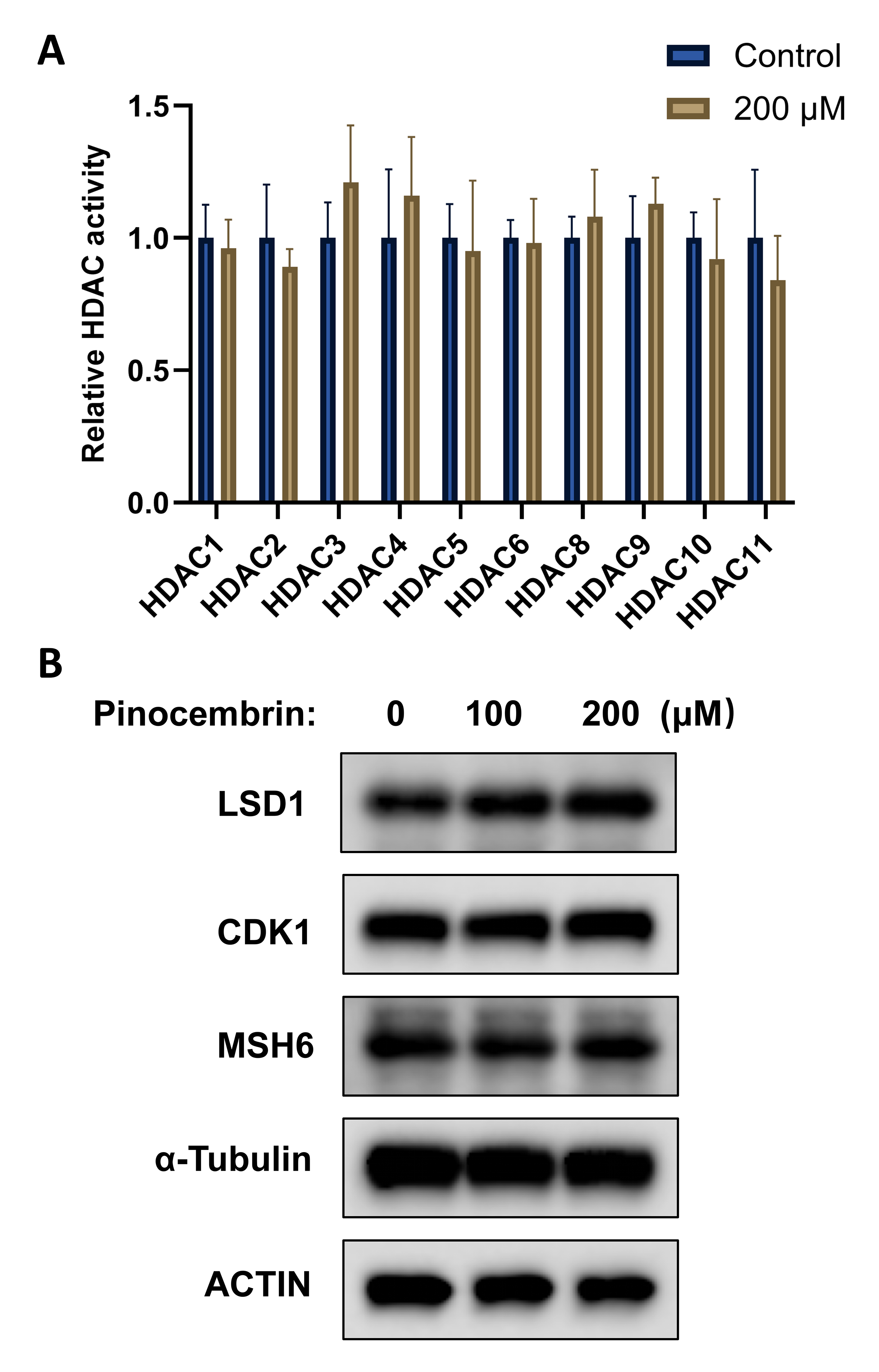

Supplement: Supplementary file 15 — Supplementary Material 15: Figure S10. Pinocembrin does not exert a general suppressive effect on HDAC activity. A. T24 cells were treated with 200 μM concentration of Pinocembrin, HDAC activity was assessed in cells with different HDACs (HDAC1-6 and 8-11) expression levels using an HDAC Activity Assay Kit. B. The effect of Pinocembrin on the expression of known non-histone substrates of other HDACs, such as LSD1, CDK1, MSH6, and α-Tubulin, were studied through Western blot. Data are mean ± SD, n= 3. [file 13046_2025_3585_MOESM15_ESM.tif]
